# Remotely seeing is believing: how trust in cyber-physical systems evolves through virtual observation

**DOI:** 10.1098/rsos.260220

**Published:** 2026-10-28

**Authors:** Zhi Jin, Kurt Xiao, David Hyde

**Affiliations:** Institute for Convergent Software Integrated Systems, Vanderbilt University, Nashville, TN, USA

**Keywords:** trust, cyber-physical systems, video streaming, user study

## Abstract

We study how passive virtual observation of cyber-physical systems (CPS) can affect human trust in those systems. We perform this study via a newly developed novel web application that enables researchers to show pre-recorded or live video feeds to groups of users in a synchronized fashion. Users are able to provide real-time feedback on these videos via affect buttons and a free-form chat interface. We evaluate our application via a quantitative user study (N≈80) involving videos of CPS, such as autonomous vehicles (AVs), performing positively or negatively. Using data collected from user responses in the application, as well as customized survey instruments assessing different facets of trust, we find that human trust in CPS can be affected by remotely observing the behaviour of such systems—without ever encountering them in person.

## 1. Introduction

Cyber-physical systems (CPS) are network-connected devices and systems with physical presence or form. CPS encompass a wide range of systems across scales of size and complexity; examples include autonomous cars, smart homes, pedometers and smart grids. Technologies in this category are fundamentally altering the human experience and the ways in which everyday citizens act and interact. Given their importance, the study of CPS is an active area of research—including their modelling [1], design [2,3] and security [4,5]. In the past few years, increasing attention has been given to human factor aspects of CPS, such as interface design and evaluation [6–10].

As with any technology, the human factor of trust is essential—if not for the utility of a technology, then at least for its widespread adoption. For example, trust (or lack thereof) in autonomous vehicles (AVs) is well known to influence their adoption [11]. Trust and acceptance are also known to be related in the case of industrial CPS, such as nuclear power plants (PPs) [12–15]. Trust—which must be carefully defined and measured—has also been shown to be a dynamic quantity, and one that can be permanently altered when a CPS performs contrary to a user’s expectation [16].

Given that trust is a potentially malleable quantity, the ultimate goal of the research in this paper is to provide methods that may increase trust in CPS. Increasing trust in CPS is potentially important for a number of reasons. For example, as AVs become safer than human-driven vehicles, trust in CPS leading to AV adoption could prevent accidents and hence save lives. Similarly, given nuclear PP clean emissions profile [17], increasing trust in those CPS can contribute to saving our planet from the effects of accelerated climate change. We view these examples alone as sufficient to motivate our investigation.

In this paper, we study the development of user trust in CPS through a novel ‘virtual laboratory’ for human trust. Unlike conventional participatory studies where users directly manipulate, exchange information with, or control systems, our research investigates how trust develops when participants *remotely* observe CPS *passively* through curated video clips. Our web interface enables users to provide feedback on videos of CPS they watch via affect buttons and a free-form textual chat interface. The laboratory is configured so that a group of participants may all be shown the same videos simultaneously, and participants can see each other’s feedback in real time, replicating a traditional focus group. We deliberately retain this social visibility as part of the studied setting: online video today is increasingly consumed in socially embedded contexts—live streams with public chat, comment overlays and visible audience reactions—so our study examines trust formation under passive observation in such a setting (§6 discusses the implications of this design choice).

Our paper also presents customized survey instruments, based on established trust models and measurements typically used in direct interaction scenarios, to study trust formation in this passive observational context. We use our virtual laboratory and these trust instruments to address the fundamental research question: how does mere remote observation of system performance, in a socially embedded viewing setting, influence participants’ trust development over time? Through a quantitative user study of approximately 80 participants, we find statistically significant evidence that virtual observation of CPS performing well or poorly affects human trust in the underlying CPS.

The main contributions of this paper are:

— The design and implementation of a web application for studying human trust in CPS in a controlled environment using pre-recorded videos or live streams alongside affect buttons and a textual chat interface.
— Results from a user study, using this application, that include statistically significant findings for trust being influenced by remote, passive observation of CPS in a socially embedded viewing setting.
— A survey instrument, based closely on prior works, specialized for evaluating learned trust in the context of CPS.

Finally, before proceeding, we address the question of why online videos are an appropriate or especially useful object of study for trust in CPS. Since many humans are unable to practically observe various CPS in person (such as at a nuclear PP), it is necessary to present them with a remote proxy such as an online video in order to show them the operation or performance of the system. Moreover, the average American adult watches at least 5 hours of television and video per day [18]—almost 30% of their waking hours, on average [19]—so videos are a particularly important and practical means by which one can reach the average person. Furthermore, since people are spending perhaps 3 hours per day on social media [20], which is increasingly trending towards short-form video content (TikTok, Instagram Reels, YouTube Shorts, etc.) [21,22], we are particularly inclined to study how relatively brief exposure to videos of CPS can potentially influence longer-term trust. Thus, our motivation is grounded in the present practical realities of the human experience and the increasing prevalence and influence of media and videos.

## 2. Related work

Trust is an extensively researched concept in human–computer interaction (HCI). From data visualization [23] to social media [24,25] and, of course, to AI and human–AI interactions and teaming [26,27], HCI researchers recognize the paramount importance—and, in some cases, perils [28]—of trust in computer and digital systems.

However, as works like Zhang *et al.* [25] and Wischnewski *et al.* [29] point out, trust is not, on its own, a well-defined quantity, and one must rigorously define specific dimensions of, and metrics for, trust in order to conduct quantitative experiments and measure the effects of interventions. In this section, after briefly situating the present work among the existing HCI literature, we focus on related work on defining and measuring trust in §2.1, which motivates the interfaces and survey instruments we ultimately design and use for our quantitative user study.

*Trust and videos.* The present work performs interventions by showing real-time streaming or pre-recorded online videos to users. In their classic works, Bos and colleagues [30,31] assessed how interpersonal trust develops when users communicate with each other over videoconferencing (akin to our streaming modality) and find similar levels of trust as with in-person experiments, though they identified delayed trust development in the virtual setting. These insights inspired our central research question, whether and how trust dynamics evolves when humans remotely observe CPS rather than interact with other humans. Nguyen & Canny [32] built upon Bos *et al.* [31] and found that eliminating spatial distortions in video feeds improved trust. More recently, Pan & Steed [33] evaluated whether users trusted videos, robots or avatars more when seeking expert advice. Horn & Severus [34] considered how tutorial videos (e.g. in music) could build user trust. In another line of inquiry, works like Vaccari & Chadwick [35] have studied the detrimental effect of deepfake videos on human trust. Related to the present work, Avetisian *et al.* [36] primed experimental subjects into low- and high-trust groups and then showed them a video of AV performance and predicted emotional responses based on the priming. In our study, we ask somewhat of the opposite question, using real-time measurements of emotion and pre-/post-survey instruments to assess how videos affect trust development.

*Trust and CPS.* In the HCI literature, AVs are perhaps the most studied CPS in recent years when it comes to human trust. For instance, Fu *et al.* [16] used a physical immersive AV simulator with a physical ‘trust dial’ to measure how users’ trust changed over time as the AV simulator behaved well or poorly. In their study, where users physically interacted with (and at times, operated) an AV, the authors found that trust can be permanently damaged as soon as the AV performs poorly. Along another dimension, Kaufman *et al.* [37] used machine learning to identify the most pertinent human factors that influence trust in AVs. In a similar modality to the present study, Colley *et al.* [38] showed one video of an AV to participants across different groups, with each group seeing different semantic segmentation visualizations overlaid on the video. Factors such as trust were measured with instruments including a German version of the Trust in Automation scale of Jian *et al.* [39]. Unlike Colley *et al.* [38], our study shows videos of different CPS operating under differing conditions, and we leverage customized survey instruments, affect buttons and free-form text answers to build our measurements of trust. On the modelling front, Gil *et al.* [40] developed a design theory for human-in-the-loop autonomous CPS, such as AVs, that aims to formalize many of the considerations found in human–AV and human–CPS trust papers. Finally, Rahman [41] presented a human–robot interaction framework where *mutual* trust between the human and robot (CPS) is modelled and updated during the course of a task.

### 2.1. Defining and measuring trust

#### 2.1.1. Dimensions of trust

To address and attempt to unify the diverse definitions of trust in the literature, Razin & Feigh [42] (see also [43]) compared, mapped and analysed the most frequently cited and best-validated trust survey instruments, ultimately proposing a structured model for trust that includes three primary dimensions:

*Dispositional trust* refers to an individual’s general propensity to trust systems prior to encountering any specific situation or interaction. Although dispositional factors tend to persist over time, they can be influenced by long-term experiences, demographic variables and personality traits [42]. Hoff & Bashir [43] extended the concept to human-automation contexts, framing dispositional trust as an individual’s general tendency to trust technological systems. More recently, Razin & Feigh [42] identified faith in technology as a key antecedent belief that influences expectations and emotional responses towards emerging technologies.

*Situational trust* reflects the extent to which trust is formed or adjusted based on characteristics of the immediate context. Situational trust changes based on momentary environmental cues, user expectations and conditions of the task at hand. Users encounter these situations when they must rely on the incomplete knowledge of a system’s intent and capabilities [44,45]. Thus, in these contexts, situational trust emerges through the interaction between the trustor (the user) and the trustee (the system), with trust judgments shaped by perceived system performance, reliability and contextual risk [43,46].

*Learned trust* refers to the trust that forms and adapts based on an individual’s direct past experiences with a specific system or through ongoing system interactions. Unlike situational trust, which is shaped by the immediate environment and contextual factors, learned trust develops from accumulated knowledge about a system’s performance, behaviour and reliability over time [47].

The present work measures each of these dimensions of trust. As participants in our study arrive with pre-existing tendencies that influence their initial interpretations of system behaviours, measuring dispositional trust prior to the observation experience allows us to distinguish between trust shifts attributable to inherent predispositions and those resulting from the video portrayals of CPS performance. Our study also leverages situational trust to examine how immediate environmental cues and observed system behaviours across varying scenarios shape participants’ perceptions. By evaluating key situational antecedents, we capture how users’ initial trust perceptions are formed and how these perceptions recalibrate as new contextual information unfolds during passive observation. Finally, we leverage learned trust to examine how participants’ perceptions of the system evolve throughout the observation experience. By assessing participants’ initial confidence and tracking shifts in response to observed system behaviours, we capture both the influence of prior experiences and the development of new impressions during real-time engagement.

#### 2.1.2. Trust calibration

In the user study discussed in this paper, participants observe CPS behaving under varying conditions over time. Thus, participants undergo *trust calibration*, which Muir [48] defines as the process of aligning a user’s trust with a system’s actual capabilities, limitations and behaviour. Rather than viewing trust as a fixed attitude or general belief about a system, Muir [48] emphasizes that trust should be shaped over time through repeated observations of how a system behaves.

Wischnewski *et al.* [29] expanded this perspective by identifying distinguishing factors of trust calibration interventions, including several relevant to our work. Our study performs endo calibrations—calibrations that take place during the interaction itself (rather than before or after, such as priming), allowing users to update their trust based on how a system performs in real time. Warranted calibration is relevant for CPS as it refers to strategies that aim to support trust judgments that accurately reflect the system’s actual performance. Warranted interventions are grounded in verifiable characteristics of the system, such as its accuracy or robustness, rather than relying on superficial cues like aesthetics or reputation. Our study uses static calibration: interventions that are fixed and uniformly applied across all users (not adaptive based on user-specific behaviour or real-time indicators of over- or under trust). Finally, our study performs performance-oriented calibration, which involves focusing on what the system does—its reliability, accuracy or task success—without necessarily revealing how those outcomes are produced. This is particularly relevant for complex CPS such as PP. We refer to [29] for further details on the taxonomy of trust calibration.

#### 2.1.3. Related trust instruments

Comprehensive meta-analyses and systematic reviews conducted by Wischnewski *et al.* [29], Razin & Feigh [42] and Krausman *et al.* [47] highlight the necessity of carefully aligning assessment instruments with their specific context of application. Specifically, Wischnewski *et al.* [29] advocated for the implementation of complementary methodological approaches that integrate both self-report measures and behavioural indicators to accurately capture fluctuations in user trust perceptions. Furthermore, they emphasize the importance of trust assessment before, during and after HCI. Our study takes advice on both these fronts, conducting pre- and post-surveys as well as collecting behaviours and self-reported answers during our interventions.

Several established instruments capture different dimensions of user trust. Jian *et al.* [39] developed a scale to examine how system characteristics influence users’ perceptions of trust. They also noted that assessing individuals’ general inclination to trust automated systems could serve as a valuable anchor for understanding how trust develops and changes over time. Though well-established, Jian *et al.* [39] do not factor trust into dimensions such as dimensional, situational and learned trust. Partly addressing this, Schneider *et al.* [49] created an instrument for measuring dispositional trust that captures stable cognitive and affective propensities towards system trust. We use their instrument to assess dispositional trust in our study.

McKnight *et al.* [50] presented a structured trust framework and accompanying questionnaire that distinguishes between disposition to trust, institution-based trust, trusting beliefs and trusting intentions. We found their trust dimensions to be equivalent to the dispositional, situational and learned factorization of trust discussed above: disposition to trust corresponds to dispositional trust; institution-based trust is situational, and trusting beliefs and trusting intentions map onto learned trust. Although their instrument does capture different dimensions of trust, it is specifically designed for e-commerce, which made it difficult to directly adopt for our study on CPS.

For our purposes, Lee & See [44] combine the advantages of Jian *et al.* [39] and McKnight *et al.* [50]. The authors present a trust model focused on trust in automation (which is sufficiently similar to CPS) that also divides trust into various measurable dimensions that can be measured. Their trust framework considers an assortment of descriptors that contribute to categorical characterizations of the performance (how reliably and competently the system achieves its goals), process (how understandable and appropriate its underlying operations appear) and purpose (the extent to which the system’s intent and use align with the user’s goals and values) of an automated system. We adapted this framework to construct our questionnaire and assess participants’ learned trust. Importantly, these three dimensions also underscore the formation of situational trust, providing the informational basis through which users recalibrate their trust in response to contextual cues about system performance, process and purpose. We further derived our real-time qualitative trust questions from this framework, where each question corresponds to one of the three bases, enabling us to capture trust dynamically during CPS observation.

While many validated instruments—such as those developed by Jian *et al.* [39], Schneider *et al.* [49] and McKnight *et al.* [50]—explicitly reference trust in their item wording, our study intentionally avoids using the word altogether. This decision reflects a design choice aimed at reducing priming effects and encouraging participants to respond based on their perceptions and judgments of the system’s behaviour, rather than aligning their answers with preconceived notions of what trust entails. This allows us to explore how these underlying constructs develop through observation, without shaping participant interpretation through the language of trust itself.

Finally, we note that constructing a context-matched instrument from validated item pools, rather than adopting a single legacy scale unmodified, is consistent with the recommendations of recent reviews of trust measurement [51,52] and addresses documented concerns about the applicability and bias of standard instruments outside the direct-interaction settings for which they were developed [53–55]. Section 4 details the provenance of our instruments and the validity evidence we provide for them.

## 3. Virtual laboratory design and study capabilities

Our study platform is a web application that connects participants and researchers in real-time sessions. A researcher may create various sessions using our application and assign different subjects to different sessions.

Each session involves watching a pre-recorded video or a live video stream. During a session, subjects are able to interact via a free-form text chat interface.^1^ At specific times chosen by the researcher when setting up the session, subjects are prompted to click affect buttons or to answer qualitative question prompts in the chat. The inspiration for our platform was to build a system that enables workers or study participants to complete tasks that require synchronized effort and that may require collaboration (e.g. [57]); for example, one may wish for study participants to collaboratively analyse behaviours observed during a live video feed of a complex CPS. These capabilities are in contrast to platforms like Amazon Mechanical Turk [58], which do not facilitate real-time or collaborative tasks or studies.

Though not utilized during the study in §5, we also built support in our platform for recording where subjects click within a video during the course of a study. For instance, one could ask participants to watch a sports game, and to click when and where they observe fouls or rule violations.

Our virtual laboratory automatically records, organizes and saves all data collected from users during a session into CSV files for later analysis. All session users must authenticate to the web application with their email address, so the system can consistently associate logs and records with the correct user.

The laboratory’s web front-end is built using React, HTML and CSS. Server-side infrastructure is built using Node.js, a JavaScript runtime environment; thus, both the front-end and back-end are primarily in JavaScript. Back-end code is written using the model-view-controller architectural pattern for ease of maintainability and scalability. A standard MySQL database stores session and user information. Finally, the Socket.IO JavaScript library is heavily used to enable bidirectional communication between clients and servers via WebSockets. All infrastructure is hosted in the cloud (AWS) and can run using only small or medium instance types.

Figure 1 illustrates the typical user flow through the web application (top), for subjects or researchers.

**Figure 1.**
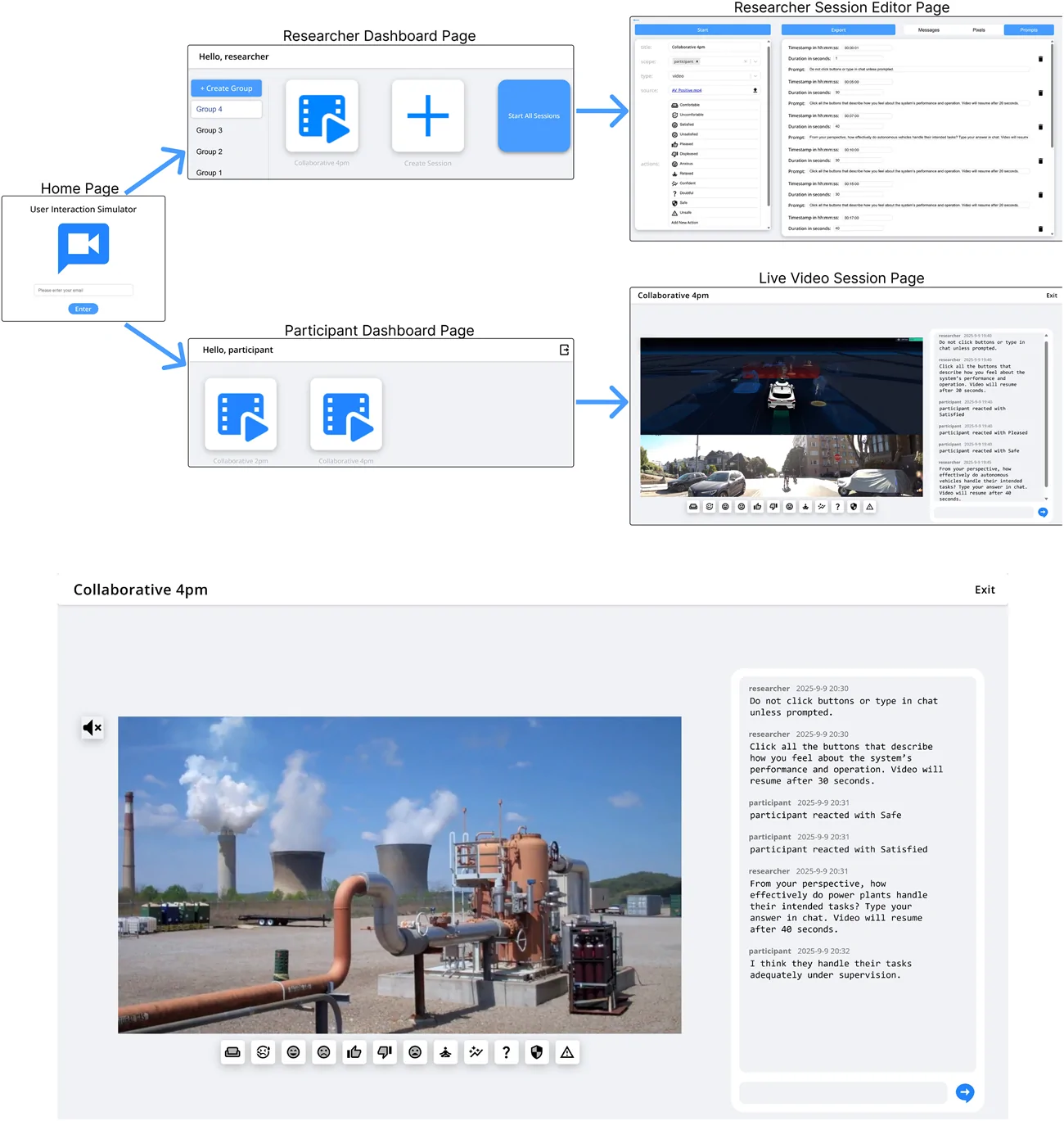
*(Top)* A flow chart of using our virtual laboratory for trust measurement. After signing in to the web application, participants can join any study sessions to which they are added by researchers. Researchers can create new study sessions, during which our interface allows specifying what video or content to display to participants, what affect buttons to show and what prompts to ask participants (and when). Participants and researchers can then conduct and participate in a session, respectively. *(Bottom)* A close-up of the study session view for a participant. Here, the main portion of the screen plays a video of power plant (PP) operations. Affect buttons below the video enable the participant to specify their reactions to content. A free-form text chat interface allows users to answer qualitative questions from researchers, and to see other participants’ reactions and responses.

The bottom panel of figure 1 offers an example of the session view for a participant, including the video content, the affect buttons (see also figure 2) and the free-form text chat interface.

**Figure 2.**
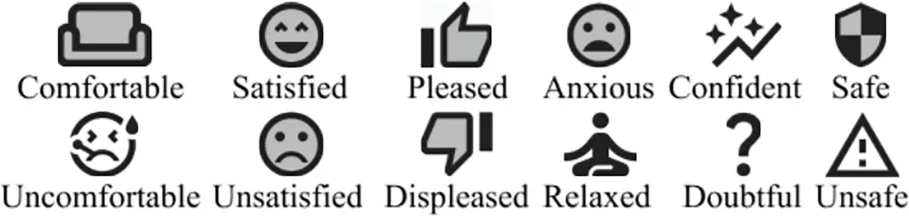
Affect buttons used in our study. Here, the buttons are arranged in bipolar pairs.

## 4. Trust instrument construction and trust scores

As motivated in §2.1, we design trust instruments—including pre- and post-surveys, as well as endo calibrations—that aim to measure dispositional, situational and learned trust. To measure dispositional trust, we present study participants with a pre-survey that is the dispositional trust instrument of Schneider *et al.* [49]^2^ with the word ‘technology’ replaced with ‘CPS’ throughout.^3^

Aside from the dispositional questions in the pre-survey, our pre- and post-surveys are identical. We leverage them to assess learned trust relative to baseline values by computing the difference in values between the post- and pre-surveys. The surveys consist of 18 items based on keywords identified in [44] and well-corroborated by established trust research [39,60–67]. Each item is measured on a standard five-point Likert scale. For ease of exposition, we present each item, along with supporting quotations from the literature, in appendix A.

Because trust is the central outcome variable of this work, we briefly discuss the provenance, motivation and justification for this 18-item instrument, which we call the CPS trust observation scale (CPS-TOS). CPS-TOS is best characterized as a systematically adapted instrument: a newly assembled questionnaire whose items are adaptations of items from established, validated trust scales, selected and organized according to the performance/process/purpose framework of Lee & See [44], with each item’s referent rewritten for CPS. It is thus neither a single previously validated scale adopted wholesale, nor an ad hoc questionnaire constructed de novo; appendix A documents, item by item, the validated source items on which each CPS-TOS item is based.

We deliberately did not fully adopt a single established instrument, for three reasons. First, to the authors’ knowledge, no validated trust instrument exists for our setting: established scales were developed for settings in which the respondent directly operates, relies upon or transacts with the system—e.g. Jian *et al.* [39] for operators of automated systems, McKnight *et al.* [50] for e-commerce and Körber [61] for automated driving—whereas our participants passively and remotely observe CPS without interacting with them. Items presupposing direct reliance are not obviously applicable in this setting; indeed, Chita-Tegmark *et al.* [53] show empirically that when respondents are given the option, they judge many items of standard trust questionnaires ‘not applicable’ to the specific system being rated, and Gutzwiller *et al.* [54] report evidence that the widely used scale of Jian *et al.* [39] exhibits positive bias. Second, there is no single ‘gold standard’ instrument to adopt: reviews of trust measurement in automation document dozens of coexisting instruments and recommend selecting or tailoring measures to the system and study context [51,52]; a systematic review found that roughly 41% of trust-in-automation studies employ researcher-constructed measures [52], and even the scale of Jian *et al.* [39] is itself routinely modified in items, referents and formats to fit new contexts [55]. Third, we required an instrument that avoids the word ‘trust’ to reduce priming.

Our construction procedure follows best-practice recommendations for scale adaptation—retaining the substance of validated items, documenting every change against the source items and reporting psychometric evidence for the adapted form [68]. Scale adaptation of this kind is common, accepted measurement practice: Heggestad *et al.* [68] found that nearly half of the scales used in leading organizational science journals are adaptations of existing measures, and Jessup *et al.* [59] adapted the scale of Jian *et al.* [39] analogously by replacing its referent. With respect to validity evidence for the adapted form [69], content validity is supported by the systematic grounding of every item in the framework of Lee & See [44] and in one or more validated source instruments (appendix A); internal consistency is high for the full instrument and for each of its three subscales (table 1), and, as reported in §5.1, the instrument behaves as a valid trust measure should, with scores moving in opposite directions under positive versus negative CPS observation (under both affect button responses and sentiment-scored free-text responses). We acknowledge that full psychometric validation of CPS-TOS—e.g. factor-analytic replication and test–retest reliability estimation in an independent sample—remains future work (§6.1).

**Table 1.** Cronbach’s α values for assessing internal consistency of our survey instruments for learned and dispositional trust. For the learned trust instrument (used for both pre- and post-surveys), we also analyse components of the instrument corresponding to performance, process and purpose (cf. appendix A); 95% confidence intervals are generated via bootstrapping (1000 iterations). Results suggest that all instruments have α≥0.70, which is the standard threshold for internal consistency ([76, p. 265]).

instrument	Cronbach’s α	95% confidence interval
learned trust (full)	0.937	[0.924,0.947]
learned trust (performance)	0.902	[0.877,0.920]
learned trust (process)	0.793	[0.753,0.823]
learned trust (purpose)	0.877	[0.850,0.898]
dispositional trust	0.798	[0.697,0.855]

Regarding situational trust, we perform endo calibrations, prompting study participants to provide free-form text and affect button responses at specific times during a study. Free-form text responses are collected via the chat interface in our application, and our web interface presents a pop-up for users to directly click on affect buttons. For qualitative questions, we adapt our prompts from Lee & See [44]. For the affect buttons, the emotions we allow users to select are visualized in figure 2. We adopt these emotions from the Geneva Affect Label Coder of Scherer [70, table 4]. Given the focus of the present study on CPS, we prune several categories that are unlikely be the dominant emotion a study participant would express towards a CPS during the study, such as longing and lust. We also seek to ensure that each emotion appears as a bipolar pair; for affect categories lacking a clear polar opposite in [70], we consulted several highly cited works [71–74] to add appropriate opposites. Given the importance of safety to CPS, we also add safe/unsafe as an affect pair to our instrument.

In analysing our results (§5.1), we seek to understand how overall trust evolves for study participants. To aid in this analysis, we define a dispositional trust score that can be assigned to each study participant. The dispositional trust score is the sum of the numerical values of the responses to each of the five-point Likert-scale questions that a participant provides on the dispositional trust survey—noting that we invert the scores for any inverted (negative) questions. We similarly define and compute a latent trust score based on participants’ responses to the pre- and post-study survey instruments for each study session. One consequence of defining these trust scores is that latent trust scores, or changes thereof, can be calibrated or normalized based on participants’ dispositional trust scores; for example, it should perhaps be considered less surprising that an individual with very high dispositional trust achieves a high latent trust score or experiences a large increase in latent trust.

We refer the reader to our supplementary material for complete copies of our survey instruments. To assess internal consistency of our trust instruments, as referenced above, we computed Cronbach’s α for all instruments [75] using the survey data collected through the user study in §5. The results are listed in table 1. The α values obtained suggest that the full learned trust instrument and dispositional trust instrument are both sufficiently internally consistent (α≥0.70), along with each thematic subcomponent of the learned trust instrument (performance, process and purpose).

## 5. User study

We conduct a user study using our virtual laboratory to measure the dynamics of human trust in the context of three distinct CPS: AVs, PP and robotic assembly lines (RALs). Leveraging existing real-world footage, we assemble 30-minute videos for each CPS (figure 3).

**Figure 3.**
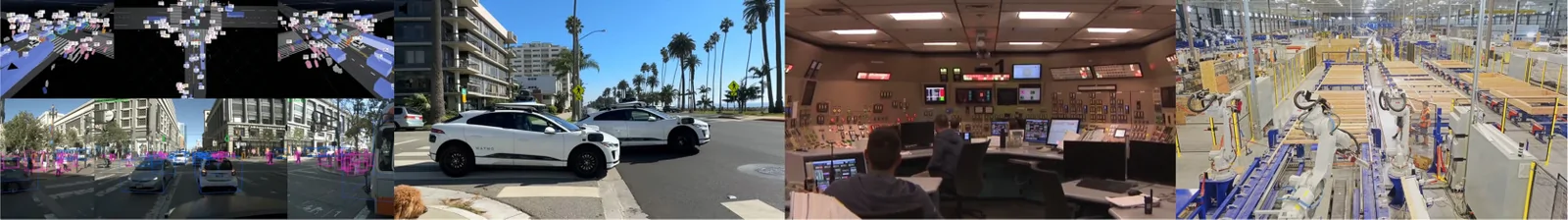
Screenshots from each of the videos different participants groups were shown during our study. From left to right: AV positive, AV negative, PP and robotic assembly lines (RALs).

These videos generally show the CPS performing well, as expected, and/or in a trustworthy way; our hypothesis (H1) is that passive observation of these videos will engender increased trust in the systems from participants. The exception is AVs, where we assemble two videos: one where AVs perform well (AV positive), and one where AVs perform poorly (AV negative). We similarly hypothesize (H2) that participants in the AV negative group will experience negative trust calibration over the course of the first session, and we further hypothesize that (H3) such participants will have lower trust scores than those in the AV positive groups on the CPS shown during the *second* session; that is, learned trust may transfer between CPS or may bias users’ general trust towards CPS in advance of the second session. Each video is generally stripped of original audio—except where necessary to understand a video segment—and an identical, neutral, instrumental soundtrack is added to each video. Appendix B provides links to each video.

We divide participants in our study into four groups, listed in table 2. We assign participants randomly, drawing without replacement from the list of participants to assign participants to each group. Each group watches either the AV negative or AV positive video during the first study session. The second study session, which is timed one week after the first (to minimize participants’ memorization of the survey questions, and to enable participants to potentially better internalize trust learned after the first session), sees participants watch either the PP or RAL videos. Despite dropout, each group ended up with approximately 20 participants.

**Table 2.** Groups for our user study. Participants were assigned randomly to the groups.

#	first video	second video	final participant count
1	AV negative	PP	20
2	AV negative	robotic assembly line (RAL)	17
3	AV positive	PP	17
4	AV positive	RAL	21

We initially recruited 83 participants who attended a first session. Participants were recruited by posting flyers around our university’s campus, as well as via social media advertisements run across USA. Our only exclusion criteria for recruitment were ensuring that participants were adults and that they were eligible to receive compensation. A $30 gift card was offered as compensation for completing two study sessions spaced one week apart. After the first session, the study suffered minor participant dropout; 75 participants completed both sessions. Nonetheless, pre- and post-survey data from participants who completed only the first session were still able to be used for certain analyses in §5.1. We note that our study was reviewed by our university’s institutional review board and was approved as study #STUDY00000139.

Because our achieved sample size was determined by recruitment and retention rather than by an *a priori* power analysis, we report a sensitivity analysis, which determines the minimum detectable effect size (MDES) for a fixed α, power and n [77,78]. We follow the asymptotic-relative-efficiency (ARE) approach to power for rank tests implemented in G*Power [79]—i.e. computing non-central-t power with effective sample size $n^{\prime }=n\times \mathrm{ARE}$, where the ARE of the Wilcoxon signed-rank test relative to the paired t test is 3/π≈0.955 under normal parent distributions (and at least 0.864 for any parent distribution with finite variance) [80]. This gives the MDES at α=0.05 (two-tailed) and 0.80 power for our per-group Wilcoxon signed-rank tests (n=17–21) to be $d_{z}\approx 0.66$–0.74 ($d_{z}\approx 0.70$–0.79 under the conservative ARE bound), and for the pooled two-group analyses in session 1 (n=36–38), it is $d_{z}\approx 0.48$. The study as designed was, therefore, adequately powered to detect medium-to-large standardized effects, and, as reported in §5.1, every statistically significant pre/post effect we observe is large (matched-pairs rank-biserial correlations of 0.70–1.00, where 0.50 is conventionally a large correlation-scale effect [81]). However, we acknowledge that smaller true effects could have gone undetected at our study’s sample sizes, so non-significant results in our analyses should not be interpreted as evidence of absence (§6.1); we anticipate *a priori* power analyses for the follow-up studies outlined in §6.

Figure 4 illustrates the process of running or participating in a study session. First, users in a group all join a single Zoom meeting, which is used to speak to and coordinate participants (including providing technical support during the study). Users then complete a pre-survey—which, in the case of the first session (in all groups), included our dispositional instrument. The figure labels these surveys CPS-TOS-* to denote ‘Cyber-Physical System Trust Observation Scale’ survey, where the asterisk represents either AV, RAL or PP. We replace the words ‘the system’ in our pre- and post-surveys with ‘AVs’, ‘RAL’ or ‘PP’ depending on the session and group as appropriate.

**Figure 4.**
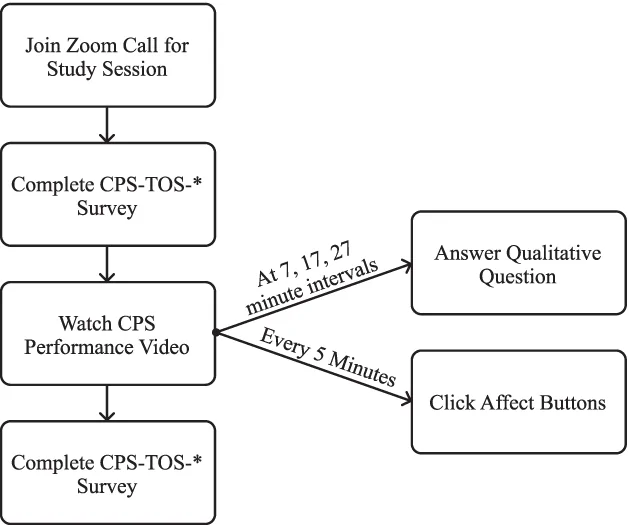
Flow chart of a user study session. Participants complete pre- and post-surveys before and after observing a CPS, during which time they are prompted to provide situational trust data at regular intervals.

After completing the pre-survey, users log in to our virtual laboratory environment using their email address. They are then able to click to join the live study session to which they were invited. Once all users in a group have joined the session, the study administrator starts playing the video in question. The video plays in a synchronized fashion across all users in the session; users are unable to start or stop the video. Every 5 minutes, the web application prompts users to click the affect button they most strongly align with based on the last few minutes of video they have witnessed. At the 7, 17 and 27 minute markers, participants were presented with qualitative prompts delivered through the application’s free-form text chat. Each prompt was adapted from Lee & See’s [44] performance, process and purpose framework to capture situational trust:

— 7 minutes (performance): from your perspective, how effectively does the system handle its intended tasks?
— 17 minutes (process): how easy is it for you to understand how the system’s operating process works in a situation?
— 27 minutes (purpose): to what extent do you believe the system is designed with people’s safety and best interest in mind?

These questions are also customized so that ‘the system’ is replaced with the CPS at hand. Each time they are prompted, users are given 30 seconds to click an affect button, or 40 seconds to answer a qualitative question. To preserve data integrity, affect buttons were disabled after the 30 second prompt window. For qualitative questions, participants were allotted 40 seconds but encouraged to continue typing beyond this period to allow adequate reflection on their observations. The free-form text chat remained open for the duration of the session so participants could complete responses at their own pace. Notably, users are able to see each other’s answers in the chat. As mentioned in §1, this socially embedded design is deliberate: it reproduces the setting in which online video is increasingly commonly consumed in practice, and our findings should be interpreted as pertaining to trust formation under socially embedded passive observation (§6.1).

After the video concludes, participants are asked to take a post-survey, which, as discussed in §4, is identical to the pre-survey. They are then dismissed from the session. An entire study session takes approximately 45 minutes to complete. The study administrator reads a prewritten script for the study session (supplementary material) to ensure timeliness and consistency between sessions and groups.

Between the two study sessions, we also asked users to complete a demographic survey (supplementary material).

Although all of the final 75 study participants completed two sessions each and all but one completed every survey (one group 1 participant missed the session 1 post-survey; see §5.1), there were instances where certain participants did not click an affect button for a particular prompt, or failed to answer a qualitative question during a session. Despite these minor limitations, our dataset is sufficiently complete to draw various statistically valid conclusions.

### 5.1. Results

*Demographics.* Of the recruited participants, approximately 70% were university students, while the remaining 30% were members of the general community. Student participants represented a diverse range of academic backgrounds, spanning STEM, social sciences, professional programs and the arts/humanities. Approximately 50% of all study participants fell within an age range of 18–26. It is known that older adults report greater distrust of technology. While distrust may not correlate with adoption [82], our population’s skew towards younger ages may bias our results towards higher trust scores. More specifically, survey evidence indicates that younger adults hold more accepting attitudes towards AVs [83], and a meta-analysis of 144 samples finds a modest, negative (though mediated largely by perceived ease of use) association between age and technology acceptance [84]; our sample’s age profile may therefore inflate *absolute* (baseline) trust levels relative to an age-representative sample. At the same time, the evidence on age and trust in automation is mixed rather than uniformly negative: experimental work has found no main effect of age on trust in automated vehicles, with older adults, if anything, maintaining *higher* trust following automation failures [85]; older adults’ expressed attitudes towards the technologies they use are predominantly positive [86]; age-related differences in adoption are substantially mediated by factors such as self-efficacy and anxiety rather than by age itself [87]. Because our principal conclusions rest on within-subject changes under randomized conditions rather than on absolute trust levels, we expect the direction of our findings to be more robust to sample composition than their magnitudes; we treat the limited representation of older adults as a limitation of the present study and discuss it further in §6.1.

By contrast, participants were somewhat well-balanced when considering ethnicity, with 23 participants reporting ‘White’, 23 participants reporting ‘Asian or Pacific Islander’ and 23 participants reporting ‘Black or African American’ (with the remaining participants selecting ‘Hispanic or Latino/Latina’ (6), ‘Multiracial or Biracial’ (5) or ‘Native American or Alaskan Native’ (1)). Aggregate plots are shown in figure 5.

**Figure 5.**
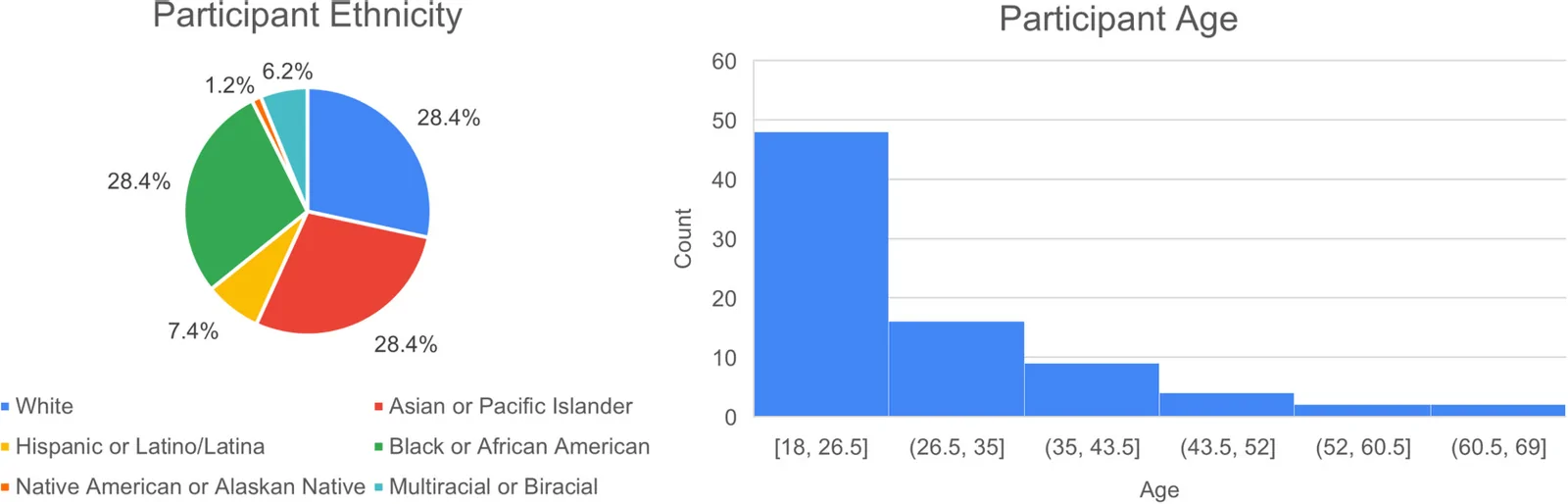
Relevant demographic factors of participants in our study, including ethnicity and age.

*Dispositional trust.* The first trust measure we analyse is the dispositional trust of study participants towards CPS (§4). Since this is a brief six-item instrument, we plot the distributions of responses to each item in figure 6. We note that participants were provided a definition of CPS in the study information sheet (supplementary material) before the study began: ‘In this study, CPS are engineered systems that combine physical (hardware) and computational (software or cyber) components to monitor and control real-world processes. Examples include smart home systems, AVs, industrial robots, automated safety systems, etc.’.

**Figure 6.**
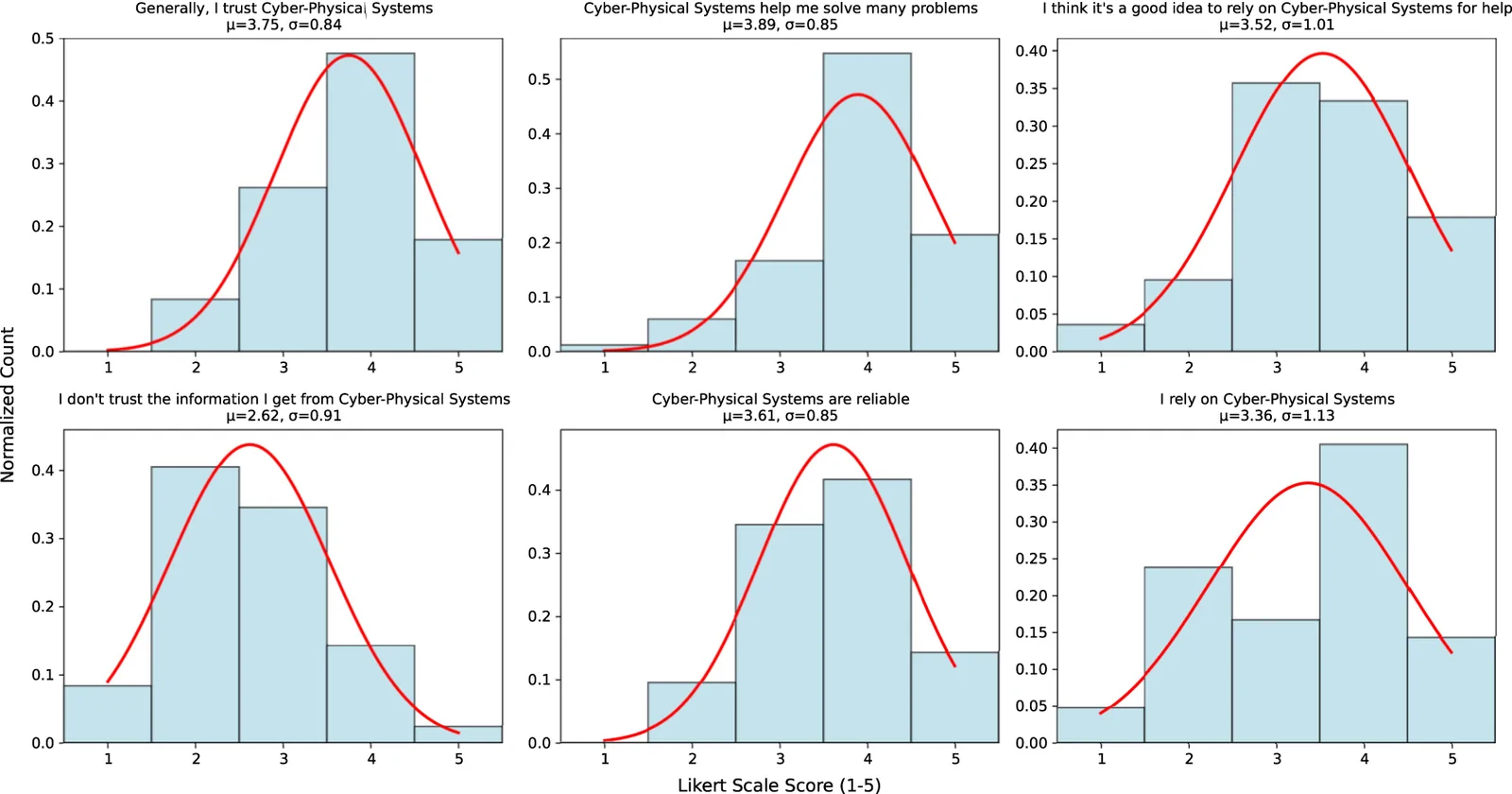
Distributions of dispositional trust factor scores. Normal distributions are fitted to the responses for each item. Study participants felt neutral or positive towards CPS on all items—though not overly positive, as the highest mean measured was 3.89 (for ‘CPS help me solve many problems’). The bottom-left subfigure represents an inverted question, where 1 indicates greatest trust.

The figure demonstrates that participants’ predisposed attitudes towards CPS were generally neutral or slightly positive. A normal distribution is fitted to each histogram (shown as red curves). Histograms are normalized in each subfigure so that the sum of histogram bars equals one in each subfigure; normal distributions are fitted to the normalized histograms.

While figure 6 shows results for all groups, we also investigated whether there were any statistically significant differences between groups (table 2). For each of the six items in the survey instrument, we performed non-parametric Kruskal–Wallis tests across the four groups, and we also performed pairwise two-sample Kolmogorov–Smirnov (KS) tests for each pair of groups. *All of these tests yielded statistically insignificant values*, even after considering the Holm–Bonferroni correction for the KS tests. Thus, we conclude that there were no significant differences in dispositional trust between the groups, which is expected and desirable since participants were assigned to groups randomly.

As discussed in §4, we also compute an aggregate dispositional trust score for each participant. Since there are no significant differences between groups, we produce one histogram of dispositional trust scores for all participants in the study (figure 7). A Shapiro–Wilk test on the data reveals a score of W=0.969 with p=0.04, implying that participants’ dispositional trust scores are normally distributed (as the figure generally suggests). Therefore, our dispositional trust score may be a reliable single score of individuals’ dispositional trust in CPS.

**Figure 7.**
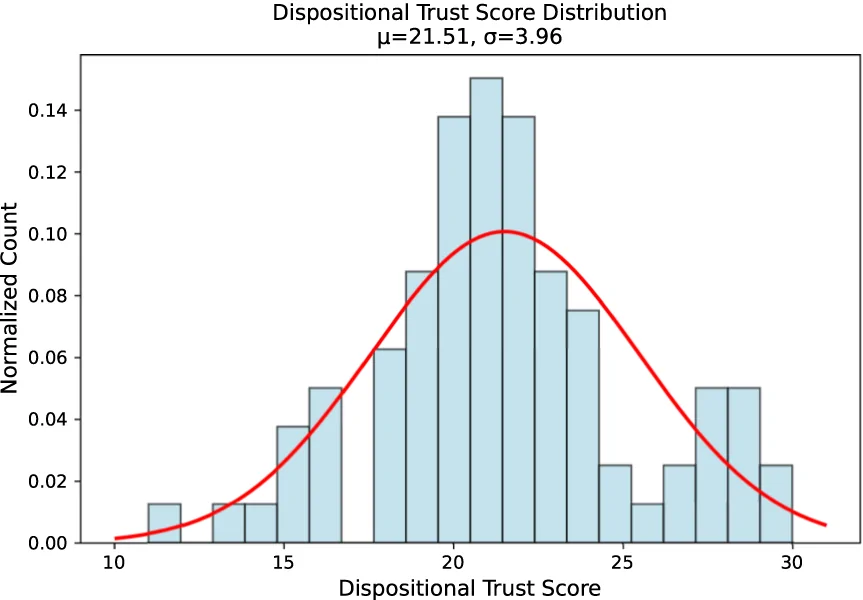
Distribution of dispositional trust scores for all participants. A normal distribution (red) is fitted to the data. A Shapiro–Wilk test affirms normality of the data. In our study, scores ranged from 11 to 30, with 30 being the maximum possible score.

*Learned trust dynamics in first session.* The first session of our study saw two of our participant groups watch the AV positive experiment—30 minutes of footage of an AV behaving well—and the other two groups watch the AV negative experiment, where various undesirable behaviours occurred with AVs (such as changing into the wrong lane or turning into traffic).

Analysis of pre- and post-survey data confirms our hypothesis that individuals’ learned trust is statistically significantly affected by watching either AV positive or AV negative. To measure the effect of the intervention, we computed a pre- and post-composite learned trust score for each participant, analogous to how the dispositional trust was computed. Figure 8 displays box plots for the pre- and post-learned trust scores for each group. Raw data points are scattered in grey. Blue boxes represent pre-session conditions, red boxes represent post-session conditions for AV negative groups and green boxes represent post-session conditions for AV positive groups. Wilcoxon signed-rank tests (table 3) confirm statistically significant differences between pre- and post-conditions for group 1,^4^ group 2 and group 4. The exception is group 3; we remark that there was an unexpected outlier in the group 3 post-survey (as is evident in figure 8), where the participant indicated the lowest level of trust for almost all survey items, despite being exposed to positive AV behaviour. Removing that participant from the pre- and post-score data changes the Wilcoxon test results for group 3 to W=44.5, p=0.074, which is on the borderline of statistical significance. See table 3 for complete statistics.

**Figure 8.**
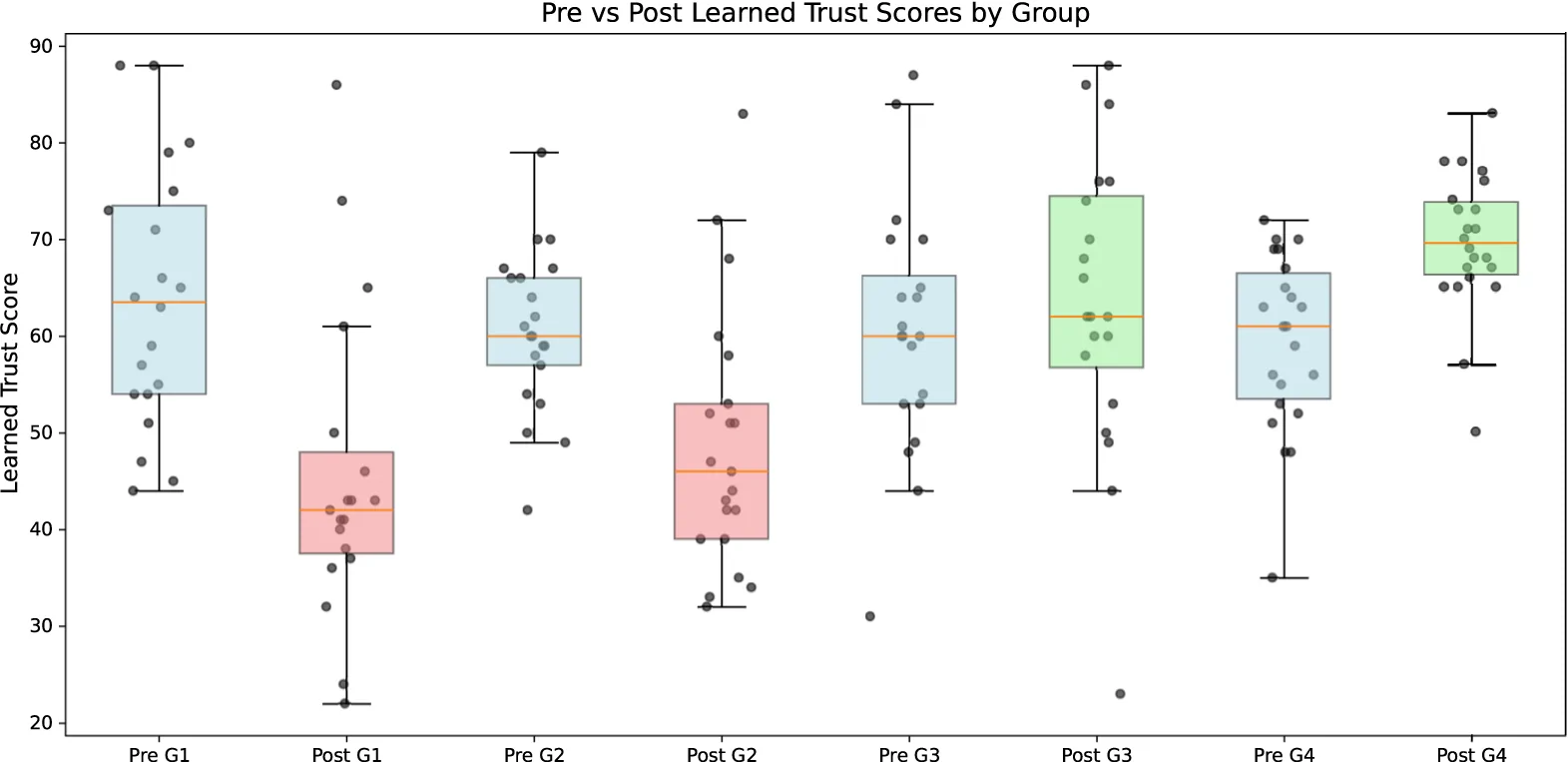
Learned trust scores, as measured by pre- and post-survey instruments, for each study group. In general, the AV negative intervention (groups 1 and 2) appears to lower trust values compared to pre-study values, whereas the AV positive intervention (groups 3 and 4) appears to generally increase trust values.

**Table 3.** Wilcoxon signed-rank test results for evaluating significance between pre- and post-conditions for session 1. The effect size (rank-biserial correlation) ranges from −1 to 1 and is interpreted similarly to Pearson’s r, with conventional benchmarks of 0.1 (small), 0.3 (medium) and 0.5 (large) effect sizes; 95% confidence intervals are estimated via bootstrapping (5000 iterations). The row labelled 3* means the data for group 3 with the extreme outlier removed (a participant who indicated almost all maximally negative trust scores, despite being shown the AV positive video). The rows labelled 1+2 and 3+4 indicate combining the two groups which were exposed to the same study condition in session 1 (leaving in the group 3 outlier).

group	statistic	p-value	significant	effect size	interpretation	95% confidence interval
1	9.00	0.0005	yes	0.904	large effect	[0.653,1.00]
2	14.5	0.0004	yes	0.875	large effect	[0.619,0.991]
3	57.5	0.131	no	0.395	medium effect	[0.026,0.801]
3*	44.5	0.0736	maybe	0.480	medium effect	[0.0526,0.867]
4	1.50	<.0001	yes	0.988	large effect	[0.928,1.00]
1+2	41.5	<.0001	yes	0.899	large effect	[0.739,0.988]
3+4	99.0	<.0001	yes	0.770	large effect	[0.548,0.935]

We note that in the first study session, groups 1 and 2 undergo the same intervention (AV negative), and groups 3 and 4 both experience AV positive. Thus, we also analyse the two group pairs combined; see figure 9 (left-hand side). In this case, the outlier effects of the participant in group 3 mentioned above are greatly ameliorated; even when leaving in that participant, Wilcoxon tests report statistically significant changes in learned trust score for the AV negative groups (W=41.5, p<0.001) and AV positive groups (W=99.0, p<0.001). See table 3 for complete statistics.

**Figure 9.**
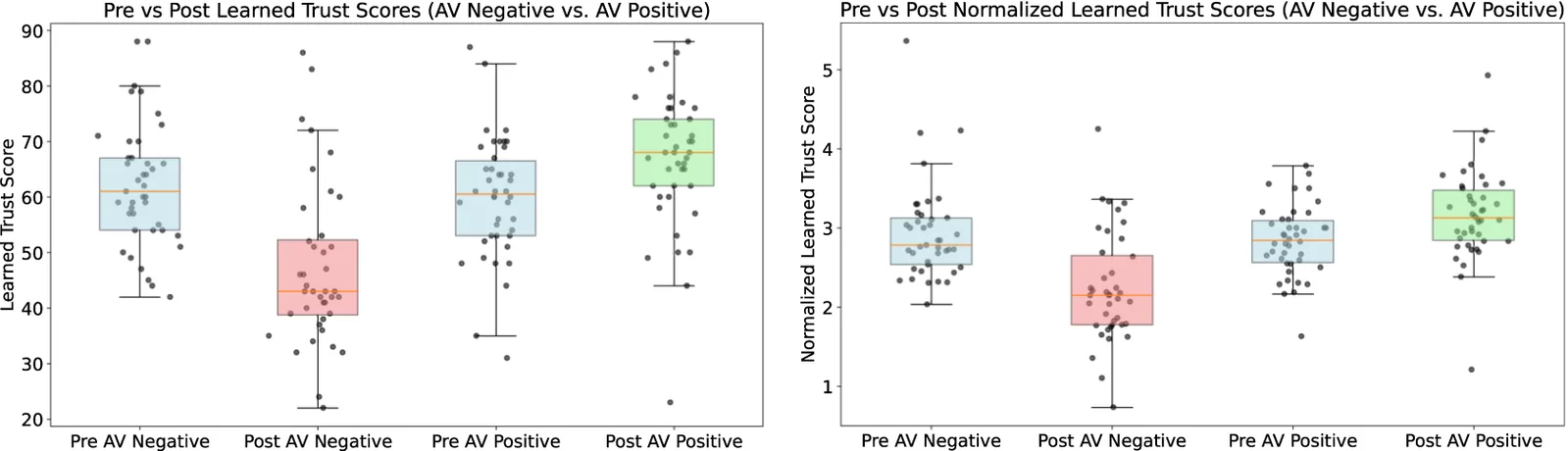
Learned trust scores, assessed pre- and post-study, for participants who experienced the AV negative intervention (groups 1 and 2) and those who experienced the AV positive intervention (groups 3 and 4).

Finally, although it is possible that learned trust scores already include the biases of participants’ varying dispositional trust, we assume for sake of argument that they do not. In such a scenario, it is useful to consider how participants’ learned trust scores evolve over the first session when normalized by their initial dispositional trust score. To assess this, we simply divide each participant’s pre- and post-learned trust scores by their dispositional trust score and re-run the statistical analyses performed above. Figure 9 (right-hand side) shows the result of this normalization, when considering AV positive and AV negative groups together. The Wilcoxon test outcomes are W=38.0, p<0.001 for AV negative, and W=111.5, p<0.001 for AV positive. Thus, whether or not normalization is applied—i.e. whether or not learned trust includes the effects of dispositional trust in this experiment—we conclude that there are statistically significant decreases (increases) in learned trust for the AV negative (positive) groups by virtue of the study intervention.

*Learned trust dynamics in second session.* We perform a similar analysis for the second session. In the second session, which occurred one week after the first, participant groups were exposed to either the PP or RAL video (table 2); thus, with four distinct conditions, we do not group AV negative and AV positive as in figure 9. Instead, analogously to figure 8, figure 10 displays the pre- and post-survey results for each of the four groups. All four groups were equivalent at the start of the second session (no statistically significant differences were observed), which was slightly surprising given groups’ varied post-survey learned trust scores after the first session. However, we note that the pre- and post-surveys for the second session used the words ‘PP’ or ‘RAL’ rather than ‘AVs’, so we conjecture that participants did not transfer their learned trust of one CPS to another, distinct CPS.

**Figure 10.**
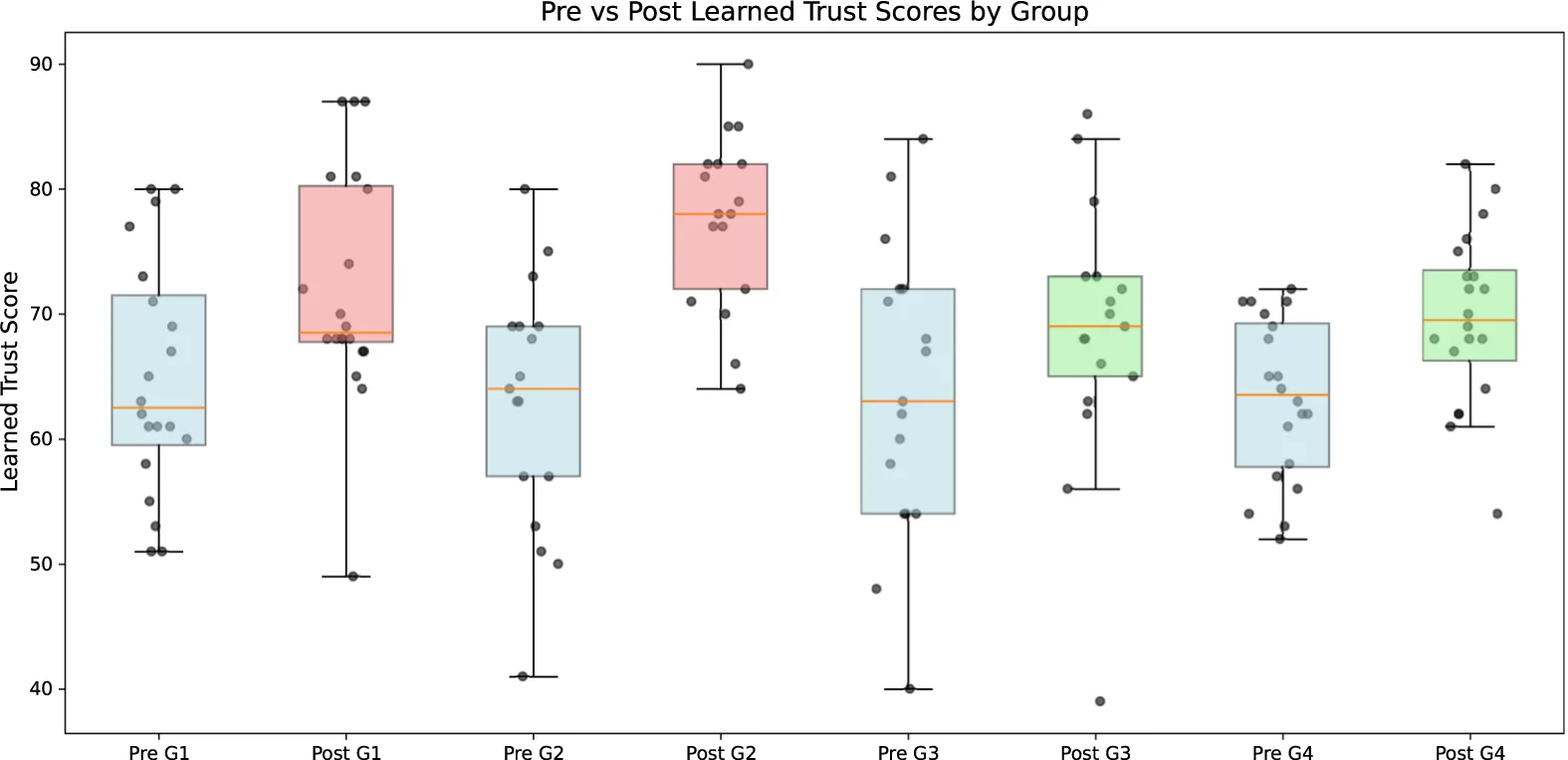
Learned trust scores, as measured by pre- and post-survey instruments, for each study group, in the second session of the study. Trust appears to increase for every study group. Generally, higher trust scores are observed for groups 1 and 2, which were first exposed to AV negative, versus groups 3 and 4, which were first exposed to AV positive.

Yet, important differences emerged when considering the post-survey data. *All* groups had statistically significantly higher trust values in post-survey data compared to pre-survey data, per Wilcoxon signed-rank tests (p<0.001). We note one caveat on these within-session increases: because every group observed a positively behaving CPS in session 2, this session lacks a negative-condition contrast, and we cannot rule out that a portion of the universal pre-to-post increase reflects repeated administration of the survey instrument rather than the observation itself (§6.1). The between-group comparisons that follow are unaffected by this concern, since all groups completed identical surveys the same number of times.

As evidenced in the figure, groups 1 and 2 (which were first exposed to AV negative) both had higher trust scores, on average, than groups 3 and 4 (which were first exposed to AV positive). Two-sided KS tests reveal that the differences between groups 1 and 3 (W=0.203, p=0.745) and 1 and 4 (W=0.2, p=0.832) were not statistically significant, although the differences between groups 2 and 3 (W=0.529, p=0.016) and 2 and 4 (W=0.556, p=0.004) were. Although only half of these comparisons allow us to definitively establish significant differences, our statistical results in conjunction with the visual evidence of figure 10 suggest that priming participants with experiencing a negative CPS led them to place even greater trust in other CPS that behaved positively. This pattern is consistent with a contrast effect: negative initial experience may lower expectations, causing subsequent positive system behaviour to exceed those lowered expectations, thereby increasing trust judgments. A future study with additional participants could help further validate this hypothesis. Complete statistics are listed in table 4.

**Table 4.** Statistical results from the second session. First, Wilcoxon signed-rank test results are shown for evaluating significance between pre- and post-conditions for session 2. The effect size (rank-biserial correlation) ranges from −1 to 1 and is interpreted similarly to Pearson’s r, with conventional benchmarks of 0.1 (small), 0.3 (medium) and 0.5 (large) effect sizes. Next, two-sided KS tests are run, comparing between each of the ‘AV positive’ groups and each of the ‘AV negative’ groups. Recall that for two-sided KS tests, the statistic value is the effect size. For all tests, 95% confidence intervals are estimated via bootstrapping (5000 iterations).

group	statistic	p-value	significant	effect size	interpretation	95% confidence interval
Wilcoxon tests						
1	28.0	0.007	yes	0.705	large effect	[0.2842,1.0000]
2	<0.001	0.0003	yes	1.00	large effect	[1.00,1.00]
3	16.5	0.0077	yes	0.757	large effect	[0.368,0.978]
4	10.5	0.0018	yes	0.863	large effect	[0.5735,1.0000]
two-sided KS tests						
1–3	0.203	0.745	no	0.203	small effect	[0.174,0.529]
1–4	0.200	0.832	no	0.200	small effect	[0.150,0.500]
2–3	0.529	0.016	yes	0.529	large effect	[0.294,0.824]
2–4	0.556	0.004	yes	0.556	large effect	[0.321,0.791]

*Situational trust dynamics: affect buttons.* As discussed in §3 and 4, we also prompted users to click affect buttons and answer qualitative questions (with free-form text responses) during each study session. These measurements are designed to assess situational trust—how individuals’ trust is affected in real time as they passively observe CPS behaviour.

We first evaluate the affect button responses. Each affect button has a polar opposite button (figure 2); we assign a score of 1 to each positive button and a score of −1 to each negative button. This can be viewed as a binary sentiment score. Figure 11 plots the mean and standard deviation of this sentiment score over time. We note that subjects were intended to click the affect buttons at 5 minute intervals throughout the study (the session automatically prompted users to do so at the appropriate times). A few participants clicked the affect buttons unprompted; these data points were removed in the figure. In addition, at each 5 minute mark, the data points in the figure are horizontally offset from each other by 0.2, for ease of readability.

**Figure 11.**
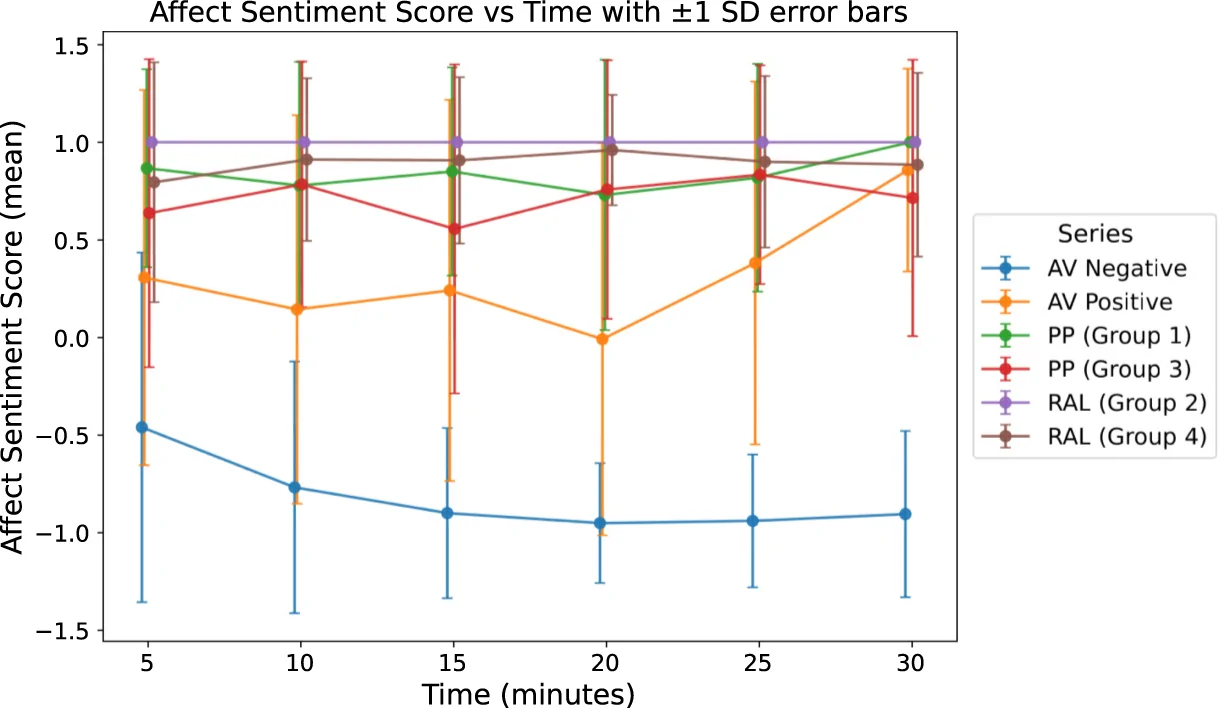
Distributions of the affect sentiment score over time during both the first and second sessions of our user study. Error bars indicate ±1 standard deviation from the mean (circles). Owing to the identical interventions applied to groups 1 and 2, and to groups 3 and 4, in the first study session, there are only two lines shown for the first session (AV negative and AV positive). One line per group is shown for the second session. In the second session, group 2 demonstrates strongly positive affect sentiment scores, while by contrast, AV negative participants during the first session record strongly negative scores.

The plot illustrates that the AV negative group quickly reaches a low sentiment score, which stays distributed quite near −1 throughout the study. By contrast, the AV positive group’s affect sentiment score tends to increase throughout the session to a distribution centred almost at +1. Owing to the study’s limited sample size, and perhaps more importantly, owing to the binary nature of this metric, the one-standard-deviation error bars on each data point are relatively large, which inhibits making conclusions about statistical significance. Another potential contributor may be recency bias; participants may have weighted their judgments more heavily on what they observed in the final moments leading up to each prompt, rather than integrating their observations across the entire preceding interval. While there is not sufficient evidence for this, we acknowledge it as a possible source of variability that may have contributed to the inflated error bars. Nonetheless, it is clear, for instance, that the AV positive and AV negative groups, while starting within 1 s.d. of each other, end up well-separated by more than 1 s.d.

Figure 11 also includes data from the second study session, where the four subject groups had differing results. Although there are visible differences, the distributions at each time overlap among all groups (again, owing in part to the binary nature of the sentiment score used here), so it is difficult to conclude that there are statistically significant differences in this situational trust metric alone. We remark that while affect buttons do indicate positive or negative sentiment, they do not measure how strongly an emotion is felt.

*Situational trust dynamics: text responses.* As mentioned in §5, we prompted users with qualitative trust questions during the study after 7, 17 and 27 minutes. To quantitatively assess these data, we also considered a sentiment score-based approach. With free-form text data, we can produce continuous sentiment score values from −1 to +1. To compute such scores, we use a transformer-based approach, leveraging the DistilBERT [88] model fine-tuned on the SST-2 dataset [89], which is available on HuggingFace as distilbert/distilbert-base-uncased-finetuned-sst-2-english. The neural network produces probability scores for the text to have positive or negative sentiment; we compute a continuous sentiment score as s=P(positive)−P(negative).

Figure 12 shows the results of this analysis and is formatted similarly as figure 11 (including filtering out responses of users not provided at the times corresponding to when questions were asked). Even with a continuous sentiment score, there are relatively large error bars, although we observe that AV negative—the only experimental condition where participants were exposed to a CPS performing poorly—consistently records the lowest mean values.

**Figure 12.**
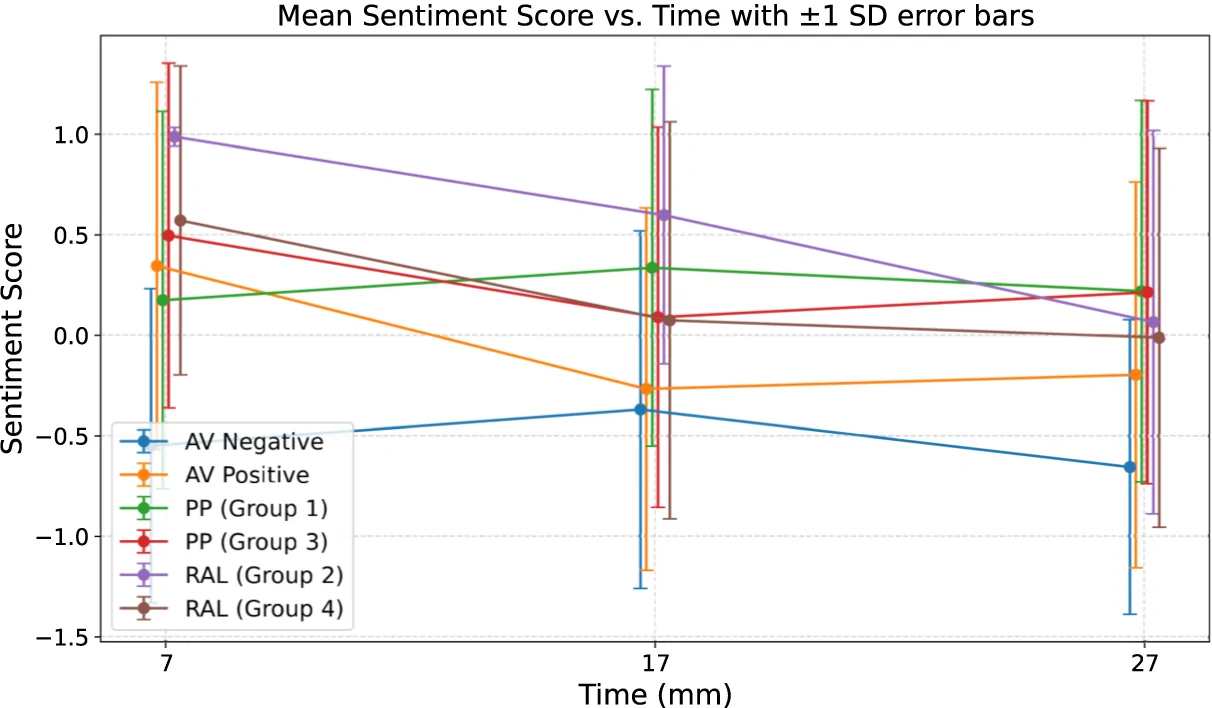
Distributions of the sentiment score over time during both the first and second sessions of our user study, based on subjects’ answers to our qualitative trust questions. Error bars indicate ±1 standard deviation from the mean (circles). Owing to the identical interventions applied to groups 1 and 2, and to groups 3 and 4, in the first study session, there are only two lines shown for the first session (AV negative and AV positive). One line per group is shown for the second session.

To check for any significant differences in the results, we performed Mann–Whitney U tests between each series. We found statistically significant differences (p<0.001) between the AV negative group and *each* other group, as well as between the AV positive group and RAL (group 2) (p<0.001)—with group 2 being the group that was exposed to AV negative during the first session. Thus, there is another piece of evidence suggesting the possibility that prior exposure to a negatively performing CPS elicits greater situational trust from participants when they are subsequently exposed to a positively performing CPS.

Overall, from analysing both affect button and qualitative question responses, and given that AV negative was the only trust intervention with negative CPS behaviour, we can conclude that situational trust was most strongly (and indeed, statistically significantly) affected by whether the trust intervention shows a CPS behaving positively or negatively. It was not significant *which* CPS was being presented to participants, which agrees with the authors’ intuition. Furthermore, in the second session, situational trust was generally not affected by which intervention subjects had experienced in the first session. Possible explanations for this include: that the week between sessions was long enough to allow subjects’ trust biases to reset; that participants’ mental models distinguished between trust in one type of CPS versus another; and that participants’ learned trust from the first session was truly an independent human factor from situational trust.

Raw, anonymized/de-identified data from the user study are available via Zenodo [90].

## 6. Discussion and future work

Our study found statistically significant evidence that human trust in CPS, as measured through our customized trust instruments, can be affected by merely watching videos of CPS—in our study, in a socially embedded group-viewing setting—without direct in-person experience. As researchers, companies and policymakers often seek to promote human trust (or mitigate factors that compromise it), we believe our work has applications across various domains where CPS are involved.

Our statistically significant results include:

— Learned trust in a CPS increased (decreased) when participants were given a trust intervention that involved passive observation of a positively (negatively) behaving CPS—regardless of the CPS being considered.
— Participants who experienced the AV negative intervention in the first session had higher post-survey learned trust values after a positive CPS intervention in the second session (significantly, at least, in the case of group 2, which was shown the RAL video in the second session).
— Situational trust did not appear to depend on any prior learned trust, but it was significantly affected based on whether the current intervention being shown to a participant was positive or negative.
— Dispositional trust was established to be statistically indistinguishable between groups, suggesting that there were no biases in our results owing to our (randomized) group assignment procedure.

Figure 13 shows these conclusions, particularly with respect to the three hypotheses stated at the start of §5.

**Figure 13.**
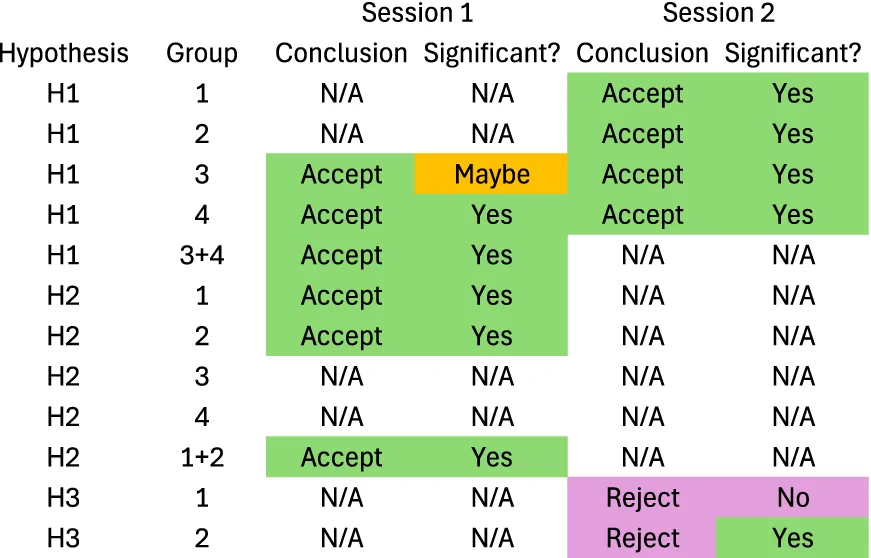
Statistical significance results for the three hypotheses in §5. H1 is confirmed, with significance, in all cases except session 1, group 3, which had one or two severe outliers. H2 is confirmed, with significance, in all cases. The opposite of H3 turned out to be true (groups 1 and 2 were *more*, not less, trusting than groups 3 and 4 during session 2), and this was significant for group 2. We note that in addition to these results, we also found that situational trust was statistically significantly affected based on whether the intervention being shown to a user was positive or negative.

### 6.1. Limitations

*Social context of observation.* In our study, participants could see one another’s affect selections and chat messages in real time. We chose this socially embedded design deliberately for ecological validity: online video today is increasingly commonly consumed in socially visible settings, such as live streams with public chat [91,92] and comment-overlaid ‘danmaku’ video [93]. Furthermore, emerging evidence indicates that trust in automated systems is itself socially transmitted within teams [94]. Our conclusions should accordingly be read as pertaining to trust formation only under socially embedded passive observation. At the same time, a long line of research shows that visible peer responses can influence individual judgments [95,96]. Indeed, trust in particular is socially propagative: individuals revise their confidence in systems not solely on the basis of direct observation but also by interpreting others’ reactions, attitudes and behaviours [97]—a dynamic further amplified through homophily, the tendency to align more closely with peers perceived as like-minded—and the perceived social pressure of important others directly shapes trusting beliefs, attitudes and intentions towards information systems [98]. Adding a communication channel to shared video viewing similarly increases emotional convergence among viewers [99]. The present data therefore cannot quantify the separate contributions of the observed CPS behaviour and of peer responses to individual participants’ trust change.

Yet, two observations suggest that peer influence does not by itself account for our findings. First, the positive- and negative-condition groups moved in opposite directions under identical peer-visibility settings, so any social influence on our results must have originated in participants’ reactions to the observed CPS behaviour rather than in a condition-independent group dynamic; peer visibility may therefore have amplified, but cannot have created, the condition effect. Second, participants did not converge to unanimity: inter-participant variance remained substantial throughout both sessions (see the error bars in figures 11 and 12), and at least one participant (the group 3 outlier discussed in §5.1) maintained strongly negative evaluations despite majority-positive peer responses. Quantifying the degree of any such amplification is discussed in §6.2.

*Repeated administration of the survey instrument.* Participants completed the same 18-item instrument four times (pre and post in each of two sessions), raising the possibility that repeated reflection on trust-related items could itself alter responses [100]. However, three considerations bound this concern. First, all groups were randomized and completed identical surveys the same number of times at the same points in the protocol, so any main effect of repeated testing is common to all groups and cancels from every between-group contrast [101,102]. Second, a retest artefact has a fixed direction for all respondents, whereas our session 1 groups moved in opposite directions according to the valence of the observed behaviour; meta-analytic estimates of pre-test sensitization are also far too small (a mean of +0.22 s.d. across outcomes, +0.29 s.d. for attitude outcomes [103]) to account for the large, condition-dependent effects we observe. Third, our primary pre/post contrasts are within-session, with administrations separated by well under 1 day, the interval regime in which sensitization effects are smallest [103]. The residual concern that pre-testing may *interact* with video exposure, which in the classical framework bears on generalization to unpre-tested populations rather than on the internal validity of our randomized between-group comparisons [101], cannot be excluded with the present design. Accordingly, we flagged in §5.1 that session 2’s universal pre-to-post increases, which lack a negative-condition contrast, may include a modest retest component. The canonical instrument for quantifying such effects is the Solomon four-group design [104], which augments a pre-test–post-test experiment with unpre-tested treatment and control arms; extending our virtual laboratory to a Solomon-type design is discussed as future work (§6.2).

*Sample size and composition.* As reported in §5, our sensitivity analysis indicates that the study was powered to detect medium-to-large effects. Smaller true effects, plausibly including subtler between-group differences in session 2, could have gone undetected, and non-significant results should not be read as evidence of absence. Our sample also skews young (roughly half of participants were aged 18–26) and was drawn largely from a university community. While this is a convenience-sampling profile common in behavioural research [105], we nonetheless acknowledge that absolute trust levels in an age-representative sample may differ from those we measured [82–84], even if the direction of change under observation proves robust [85]. Replication with larger, age-stratified and more demographically representative samples—with group sizes set by *a priori* power analysis [77,79]—is a priority for future work.

*Instrument validation.* Finally, as detailed in §4, our learned trust instrument (CPS-TOS) is a systematically adapted questionnaire that has not undergone independent psychometric validation. Confirmatory factor analysis of the performance/process/purpose structure, test–retest reliability estimation and convergent validation against established trust scales in an independent sample are planned future work; until such validation is complete, our conclusions regarding learned trust should be understood as statements about learned trust *as measured by CPS-TOS*.

### 6.2. Future work

While our study documents potentially useful findings, we believe that additional interesting work could be immediately conducted using our developed web platform as a virtual laboratory for human trust. For instance, while our study had all participants watch videos collaboratively in groups, it would be interesting to compare how trust dynamics evolves in the case where each participant watches videos and records their reactions individually, without witnessing the responses of others during the study. Such a comparison would directly address the social-influence considerations discussed in §6.1, quantifying the extent to which peer visibility amplifies (or dampens) the trust effects of observed CPS behaviour. Concretely, the immediate next study we plan with our virtual laboratory is a randomized comparison of the present socially embedded condition against an isolated-viewing condition in which peer affect selections and chat messages are hidden from each participant; because the platform already supports per-participant visibility controls, this study requires no new infrastructure. Combined with a Solomon four-group extension [104] that adds unpre-tested arms, such a design would cleanly separate the contributions of observed CPS behaviour, peer influence and repeated measurement to trust change.

Furthermore, although our virtual laboratory supports real time, live video streams or webcam feeds, the user study conducted in this paper used only pre-recorded videos. Our platform could be used to replicate studies, such as Bos *et al.* [30] or Nguyen & Canny [32], or to conduct new experiments in these contexts. With the recent rise of online streaming technologies such as Twitch, Instagram Live and TikTok LIVE, it is worthwhile to conduct further inquiry into how trust shapes and is shaped by these environments and tools.

We also note that the present paper is not a comparative analysis of different trust interventions. Yet, as future work, it would be interesting to measure the difference (if any) in effect size between trust interventions when using a passive observation system (as in the present work), a simulator (either physical [16] or virtual) or a real instance of a CPS. Intuitively, one might expect real-world interactions to induce greater trust, but works such as Bos *et al.* [30] illustrate that interactions in virtual space can yield similar trust outcomes as those in real space.

Finally, beyond the realm of CPS, we believe that the rapid proliferation and commoditization of high-quality generative AI video and deepfake tools urgently increase the importance of accurate models and taxonomies for human trust, whereby HCI researchers and practitioners can design, build and evaluate systems for a world where seeing should no longer be believing [35,106–109]. We hope that the community’s future work at the intersection of trust and video content will help, in some small part, navigate such historic shifts.

## Supplementary Material



## Figures and Tables

**Table 5.** Items in our pre- and post-survey trust instrument.

#	item	keyword or phrase [44]	use in related works
1	the system performs reliably.	reliable	•‘The system is reliable’ [39] •‘Excel is a very reliable piece of software’ [60] •‘The system works reliably’ [61] •‘What % of the time will this robot be […] reliable’ [62] •‘Please rate the robot […] reliable’ [63] •‘The system performs reliably’ [64]
2	the system operates consistently under similar conditions.	consistent	•‘The system analyses problems consistently’ [64] •‘The system performs consistently’ [66] •‘What % of the time will this robot […] act consistently’ [62] •‘Please rate the robot […] consistent’ [63] •‘The tank-spotting aid’s advice consistently helps me perform well’ [65]
3	a malfunction of the system is likely during normal use.	functional (‘What the auto- mation does’)	•‘Excel does not malfunction for me’ [60] •‘A system malfunction is likely’ [61] •‘% of time robot […] malfunction’ [62]
4	the system is capable of taking over complicated tasks.	capable (‘characteristics such as […] ability’)	•‘The system is capable of interpreting situations correctly’ [61] •‘The system is capable of taking over complicated tasks’ [61] •‘Please rate the robot […] capable’ [63] •‘I am confident about the system’s capabilities’ [61]
5	I can rely on the system to function/operate properly.	reliable	•‘I can rely on the system to function properly’ [64] •‘I feel comfortable relying on the information provided by the system’ [66] •‘The tank-spotting aid’s advice reliably helps me perform well’ [65]
6	the system responds predictably under similar conditions.	predictable (‘Predictability’)	•‘I know it is predictable’ [67] •‘The system reacts unpredictably’ [61] •‘What % of the time will this robot be […] predictable’ [62] •‘Please rate the robot […] predictable’ [63] •‘The system responds the same way under the same conditions at different times’ [64] •‘I am rarely surprised by how the system responds’ [66]
7	the system is competent at completing tasks.	competent (‘Competency’)	•‘Excel provides competent guidance (as needed) through a help function’ [60] •‘Please rate the robot […] competent’ [63] •‘What % of the time will this robot be […] incompetent’ [62] •‘The system has sound knowledge built in’ [64]
8	I understand how the system operates.	understandable (‘process […] describes how the automation operates’)	•‘I was able to understand why things happened’ [61] •‘I understand how the system will assist me with decisions I have to make’ [64] •‘I understand how the tank-spotting aid will help me perform well’ [65] •‘I will be able to perform well the next time I use the tank-spotting aid because I understand how it behaves’ [65] •‘I understand how the system executes tasks’ [66] •‘I understand the capabilities of the system’ [66]
9	it is clear how the system functions in ways that reflect integrity and compliance with standards.	integrity	•‘The system has integrity’ [39] •‘[…] has integrity, candid, sincere’ [63] •‘What % of the time will this robot […] tell the truth’ [62]
10	the system uses appropriate methods to carry out operations.	appropriate	•‘The system uses appropriate methods to reach decisions’ [64] •‘The system provides very sensitive and effective advice, if needed’ [60] •‘What % of the time will this robot…make sensible decisions’ [62]
11	it is difficult for me to identify what the system will do next.	open (‘Openness’)	•‘The system state was always clear to me’ [61] •‘What % of the time will this robot […] clearly communicate’ [62] •‘It is difficult to identify what the system will do next’ [61]
12	the system provides clear, open information about its current status and actions	open (‘Openness’)	•‘What % of the time will this robot […] communicate with people’ [62] •‘What % of the time will this robot […] openly communicate’ [62] •‘What % of the time will this robot be […] unresponsive’ [62]
13	the system behaves in ways that are appropriate for the situation.	appropriate	•‘Steps are typical of other sites’ [67] •‘Interaction is typical’ [67] •‘What % of the time will this robot […] follow directions’ [62]
14	the system is designed to act in the best interest of people.	benevolence	•‘I know it cares about customers’ [67] •‘Vendor has nothing to gain by not caring’ [67] •‘The developers take my well-being seriously’ [61] •‘Someone you can count on’ [63]
15	I believe the system will make the correct decisions when the situation is uncertain.	faith	•‘When I am uncertain about a decision I believe the system rather than myself’ [64] •‘To help me perform well, I believe advice from the tank-spotting aid even when I don’t know for certain that it is correct’ [65] •‘If I am not sure about a decision, I have faith that the system will provide the best solution’ [64]
16	the system is designed to protect users from harm or negative outcomes.	protective (‘Trustee has a positive orientation towards the trustor […]’)	•‘The system’s actions will have a harmful or injurious outcome’ [39] •‘What % of the time will the system […] protect people’ [62] •‘I feel okay using spreadsheet products because they are backed by vendor protections’ [60]
17	I believe the system is designed to operate honestly and truthfully towards users.	honesty (‘[…] to what extent the trustee has a motive to lie’)	•‘The system is deceptive’ [39] •‘The system behaves in an underhanded manner’ [39] •‘I know it is honest’ [67] •‘Vendor has nothing to gain by being dishonest’ [67] •‘What % of the time will the robot […] tell the truth’ [62] •‘sincere, candid’ [63]
18	even when I’m unsure about the system’s decisions, I have faith it is making the correct choices.	faith	•‘Even if I have no reason to expect the system will be able to solve a difficult problem, I still feel certain that it will’ [64] •‘If I am not sure about a decision, I have faith that the system will provide the best solution’ [64] •‘If I am not sure about whether to click ‘tank present’ or ‘tank absent,’ I have faith that the tank-spotting aid will provide the correct solution to help me perform well’ [65]

## Data Availability

Code for the web application is available at http://github.com/pxqg/pxqg-compile. Raw, anonymized data from the user study are available in Zenodo at [110]. Supplementary material is available online [111].

## Footnotes

^1^
While not directly related to trust, we highlight the recent work of Zhang *et al.* [56], which evaluates the utility of real-time synchronized floating comments on videos. This is similar to our system, allowing participants to see each other’s text chat messages and video reactions in real time.

^2^
This is referred to in [59] as Propensity to Trust Technology.

^3^
We note that this is acceptable practice and similar to what Jessup *et al.* [59], for instance, did in their study, replacing ‘the system’ with ‘the runner’ in the instrument of Jian *et al.* [39].

^4^
In this group, one participant completed the pre-survey but not the post-survey. In order to apply the Wilcoxon test, we removed their sample from the pre-survey data, even though that data point is included in figures 8 and 9.

## Appendix A. Trust survey instrument details

Section 4 introduces our construction of a survey instrument for measuring learned trust. To create this survey, we began with keywords or key phrases identified by Lee & See [44], and we sought to construct statements that connected each keyword to the process, performance or purpose (using the language of Lee & See [44]) of a CPS. These statements were all presented to study participants with a standard five-point Likert scale rating: strongly disagree, disagree, neutral, agree or strongly agree. For each item, we identified similar items/statements in the trust literature, beyond Lee & See [44], to properly justify the developed statements. Thus, each item in table 5 is an adaptation—with the referent changed to a CPS, following accepted scale-adaptation practice [59,68]—of one or more items from validated instruments. The right-hand column records those source items so that the provenance of every CPS-TOS item is explicit; see §4 for the full discussion of instrument provenance and validity.

In the taxonomy of Lee & See [44], reliable, functional, capable, consistent, competent and predictable relate to ‘performance’. Understandable, appropriate, open and integrity relate to ‘process’. Finally, benevolence, faith, honest and protective relate to ‘purpose’. All these factors can capture aspects of learned trust as subjects in our study passively observe CPS.

The table thus grounds our survey instrument as a reasonable interpolation of several prior trust survey instruments in the literature. In developing this instrument, the authors were encouraged by the consistency—as evidenced by the quotations in the table—of items across trust survey instruments in different domains (from consumer software, to human–robot interaction, to automated systems). We note that, similar to prior work, several of our items are inverted (i.e. presenting a negative rather a positive statement). Among other benefits, this helps detect inattentive users who may respond inconsistently with their other answers if they are not paying sufficient attention. Furthermore, attributes, such as faith and reliable, have two related items, which can also help detect inattentive users if scores on related items are wildly different.

Finally, we note that when conducting a survey for a particular CPS, we replace all instances of ‘the system’ in the survey item with the name of the specific CPS being evaluated—in the case of our user study, ‘AVs’, ‘PP’ or ‘RAL’.

## Appendix B. Experiment videos

The videos used in our study (§5) are publicly available online:

— **AV negative**: http://pxgq-videos.s3.us-east-2.amazonaws.com/AV_Negative.mp4
— **AV positive**: http://pxgq-videos.s3.us-east-2.amazonaws.com/AV_Positive.mp4
— **PP**: http://pxgq-videos.s3.us-east-2.amazonaws.com/Power_Plants.mp4
— **RAL**: http://pxgq-videos.s3.us-east-2.amazonaws.com/Robotic_Assembly_Line.mp4
