# Supplementary material for: Remotely seeing is believing: how trust in cyber-physical systems evolves through virtual observation
Source: R Soc Open Sci. 2026 Oct 28;13(10):260220. doi: 10.1098/rsos.260220 (PMC13714369; doi:10.1098/rsos.260220)
Supplement: Supplementary file 1 [file rsos.260220.zip › rsos.260220/rsos260220_si_001.pdf]

Pre-Study CPS-TOS-AV

1) What is your full name?

____

2) What is the email you used to sign up for the study?

____

In this survey, Cyber-Physical Systems (CPS) are engineered systems that combine physical (hardware) and computational (software or cyber) components to monitor and control real-world processes. Examples include smart home systems, autonomous vehicles, industrial robots, automated safety systems, etc. Please rate your level of agreement with each statement below.

3) Generally, I trust Cyber-Physical Systems

- ◯ Strongly Disagree
- ◯ Disagree
- ◯ Neutral
- ◯ Agree
- ◯ Strongly Agree

4) Cyber-Physical Systems help me solve many problems

- ◯ Strongly Disagree
- ◯ Disagree
- ◯ Neutral
- ◯ Agree
- ◯ Strongly Agree

5) I think it's a good idea to rely on Cyber-Physical Systems for help

- ◯ Strongly Disagree
- ◯ Disagree
- ◯ Neutral
- ◯ Agree
- ◯ Strongly Agree

6) I don't trust the information I get from Cyber-Physical Systems

- ◯ Strongly Disagree
- ◯ Disagree
- ◯ Neutral
- ◯ Agree
- ◯ Strongly Agree

7) Cyber-Physical Systems are reliable.

- ◯ Strongly Disagree
- ◯ Disagree
- ◯ Neutral
- ◯ Agree
- ◯ Strongly Agree

8) I rely on Cyber-Physical Systems

- ◯ Strongly Disagree
- ◯ Disagree
- ◯ Neutral
- ◯ Agree
- ◯ Strongly Agree

Please rate your level of agreement with each statement below.

9) Autonomous vehicles perform reliably
- ◯ Strongly Disagree
- ◯ Disagree
- ◯ Somewhat Disagree
- ◯ Neither Agree nor Disagree
- ◯ Somewhat Agree
- ◯ Agree
- ◯ Strongly Agree

10) Autonomous vehicles operate consistently under similar conditions
- ◯ Strongly Disagree
- ◯ Disagree
- ◯ Somewhat Disagree
- ◯ Neither Agree nor Disagree
- ◯ Somewhat Agree
- ◯ Agree
- ◯ Strongly Agree

11) A malfunction of the autonomous vehicle is likely during normal use
- ◯ Strongly Disagree
- ◯ Disagree
- ◯ Somewhat Disagree
- ◯ Neither Agree nor Disagree
- ◯ Somewhat Agree
- ◯ Agree
- ◯ Strongly Agree

12) Autonomous vehicles are capable of taking over complicated tasks
- ◯ Strongly Disagree
- ◯ Disagree
- ◯ Somewhat Disagree
- ◯ Neither Agree nor Disagree
- ◯ Somewhat Agree
- ◯ Agree
- ◯ Strongly Agree

13) I can rely on autonomous vehicles to function properly
- ◯ Strongly Disagree
- ◯ Disagree
- ◯ Somewhat Disagree
- ◯ Neither Agree nor Disagree
- ◯ Somewhat Agree
- ◯ Agree
- ◯ Strongly Agree

14) Autonomous vehicles respond predictably under similar conditions
- ◯ Strongly Disagree
- ◯ Disagree
- ◯ Somewhat Disagree
- ◯ Neither Agree nor Disagree
- ◯ Somewhat Agree
- ◯ Agree
- ◯ Strongly Agree

15) Autonomous vehicles are competent at completing tasks
- ◯ Strongly Disagree
- ◯ Disagree
- ◯ Somewhat Disagree
- ◯ Neither Agree nor Disagree
- ◯ Somewhat Agree
- ◯ Agree
- ◯ Strongly Agree

16) I understand how autonomous vehicles operate

- ◯ Strongly Disagree
- ◯ Disagree
- ◯ Somewhat Disagree
- ◯ Neither Agree nor Disagree
- ◯ Somewhat Agree
- ◯ Agree
- ◯ Strongly Agree

17) It is clear how autonomous vehicles function in ways that reflect integrity and compliance with standards

- ◯ Strongly Disagree
- ◯ Disagree
- ◯ Somewhat Disagree
- ◯ Neither Agree nor Disagree
- ◯ Somewhat Agree
- ◯ Agree
- ◯ Strongly Agree

18) Autonomous vehicles use appropriate methods to carry out operations

- ◯ Strongly Disagree
- ◯ Disagree
- ◯ Somewhat Disagree
- ◯ Neither Agree nor Disagree
- ◯ Somewhat Agree
- ◯ Agree
- ◯ Strongly Agree

19) It is difficult for me to identify what the autonomous vehicle will do next

- ◯ Strongly Disagree
- ◯ Disagree
- ◯ Somewhat Disagree
- ◯ Neither Agree nor Disagree
- ◯ Somewhat Agree
- ◯ Agree
- ◯ Strongly Agree

20) Autonomous vehicles provide clear, open information about its current status and actions

- ◯ Strongly Disagree
- ◯ Disagree
- ◯ Somewhat Disagree
- ◯ Neither Agree nor Disagree
- ◯ Somewhat Agree
- ◯ Agree
- ◯ Strongly Agree

21) Autonomous vehicles behave in ways that are appropriate for the situation

- ◯ Strongly Disagree
- ◯ Disagree
- ◯ Somewhat Disagree
- ◯ Neither Agree nor Disagree
- ◯ Somewhat Agree
- ◯ Agree
- ◯ Strongly Agree

22) Autonomous vehicles are designed to act in the best interest of people

- ◯ Strongly Disagree
- ◯ Disagree
- ◯ Somewhat Disagree
- ◯ Neither Agree nor Disagree
- ◯ Somewhat Agree
- ◯ Agree
- ◯ Strongly Agree

23) I believe autonomous vehicles will make the right decisions when the situation is uncertain

- ◯ Strongly Disagree
- ◯ Disagree
- ◯ Somewhat Disagree
- ◯ Neither Agree nor Disagree
- ◯ Somewhat Agree
- ◯ Agree
- ◯ Strongly Agree

24) Autonomous vehicles are designed to protect users from harm or negative outcomes

- ◯ Strongly Disagree
- ◯ Disagree
- ◯ Somewhat Disagree
- ◯ Neither Agree nor Disagree
- ◯ Somewhat Agree
- ◯ Agree
- ◯ Strongly Agree

25) I believe autonomous vehicles are designed to operate honestly and truthfully toward users

- ◯ Strongly Disagree
- ◯ Disagree
- ◯ Somewhat Disagree
- ◯ Neither Agree nor Disagree
- ◯ Somewhat Agree
- ◯ Agree
- ◯ Strongly Agree

26) Even when I'm unsure about the autonomous vehicles' decisions, I have faith it is making the right choices

- ◯ Strongly Disagree
- ◯ Disagree
- ◯ Somewhat Disagree
- ◯ Neither Agree nor Disagree
- ◯ Somewhat Agree
- ◯ Agree
- ◯ Strongly Agree
