# Supplementary material for: Remotely seeing is believing: how trust in cyber-physical systems evolves through virtual observation
Source: R Soc Open Sci. 2026 Oct 28;13(10):260220. doi: 10.1098/rsos.260220 (PMC13714369; doi:10.1098/rsos.260220)
Supplement: Supplementary file 1 [file rsos.260220.zip › rsos.260220/rsos260220_si_002.pdf]

Post-Study CPS-TOS-AV

1) What is your full name?

____

2) What is the email you used to sign up for the study?

____

Please rate your level of agreement with each statement below.

3) Autonomous vehicles perform reliably

- ◯ Strongly Disagree
- ◯ Disagree
- ◯ Somewhat Disagree
- ◯ Neither Agree nor Disagree
- ◯ Somewhat Agree
- ◯ Agree
- ◯ Strongly Agree

4) Autonomous vehicles operate consistently under similar conditions

- ◯ Strongly Disagree
- ◯ Disagree
- ◯ Somewhat Disagree
- ◯ Neither Agree nor Disagree
- ◯ Somewhat Agree
- ◯ Agree
- ◯ Strongly Agree

5) A malfunction of the autonomous vehicle is likely during normal use

- ◯ Strongly Disagree
- ◯ Disagree
- ◯ Somewhat Disagree
- ◯ Neither Agree nor Disagree
- ◯ Somewhat Agree
- ◯ Agree
- ◯ Strongly Agree

6) Autonomous vehicles are capable of taking over complicated tasks

- ◯ Strongly Disagree
- ◯ Disagree
- ◯ Somewhat Disagree
- ◯ Neither Agree nor Disagree
- ◯ Somewhat Agree
- ◯ Agree
- ◯ Strongly Agree

7) I can rely on autonomous vehicles to function properly

- ◯ Strongly Disagree
- ◯ Disagree
- ◯ Somewhat Disagree
- ◯ Neither Agree nor Disagree
- ◯ Somewhat Agree
- ◯ Agree
- ◯ Strongly Agree

8) Autonomous vehicles respond predictably under similar conditions

- ◯ Strongly Disagree
- ◯ Disagree
- ◯ Somewhat Disagree
- ◯ Neither Agree nor Disagree
- ◯ Somewhat Agree
- ◯ Agree
- ◯ Strongly Agree

9) Autonomous vehicles are competent at completing tasks

- ◯ Strongly Disagree
- ◯ Disagree
- ◯ Somewhat Disagree
- ◯ Neither Agree nor Disagree
- ◯ Somewhat Agree
- ◯ Agree
- ◯ Strongly Agree

10) I understand how autonomous vehicles operate

- ◯ Strongly Disagree
- ◯ Disagree
- ◯ Somewhat Disagree
- ◯ Neither Agree nor Disagree
- ◯ Somewhat Agree
- ◯ Agree
- ◯ Strongly Agree

11) It is clear how autonomous vehicles function in ways that reflect integrity and compliance with standards

- ◯ Strongly Disagree
- ◯ Disagree
- ◯ Somewhat Disagree
- ◯ Neither Agree nor Disagree
- ◯ Somewhat Agree
- ◯ Agree
- ◯ Strongly Agree

12) Autonomous vehicles use appropriate methods to carry out operations

- ◯ Strongly Disagree
- ◯ Disagree
- ◯ Somewhat Disagree
- ◯ Neither Agree nor Disagree
- ◯ Somewhat Agree
- ◯ Agree
- ◯ Strongly Agree

13) It is difficult for me to identify what the autonomous vehicle will do next

- ◯ Strongly Disagree
- ◯ Disagree
- ◯ Somewhat Disagree
- ◯ Neither Agree nor Disagree
- ◯ Somewhat Agree
- ◯ Agree
- ◯ Strongly Agree

14) Autonomous vehicles provide clear, open information about its current status and actions

- ◯ Strongly Disagree
- ◯ Disagree
- ◯ Somewhat Disagree
- ◯ Neither Agree nor Disagree
- ◯ Somewhat Agree
- ◯ Agree
- ◯ Strongly Agree

15) Autonomous vehicles behave in ways that are appropriate for the situation

- ◯ Strongly Disagree
- ◯ Disagree
- ◯ Somewhat Disagree
- ◯ Neither Agree nor Disagree
- ◯ Somewhat Agree
- ◯ Agree
- ◯ Strongly Agree

16) Autonomous vehicles are designed to act in the best interest of people

- ◯ Strongly Disagree
- ◯ Disagree
- ◯ Somewhat Disagree
- ◯ Neither Agree nor Disagree
- ◯ Somewhat Agree
- ◯ Agree
- ◯ Strongly Agree

17) I believe autonomous vehicles will make the right decisions when the situation is uncertain

- ◯ Strongly Disagree
- ◯ Disagree
- ◯ Somewhat Disagree
- ◯ Neither Agree nor Disagree
- ◯ Somewhat Agree
- ◯ Agree
- ◯ Strongly Agree

18) Autonomous vehicles are designed to protect users from harm or negative outcomes

- ◯ Strongly Disagree
- ◯ Disagree
- ◯ Somewhat Disagree
- ◯ Neither Agree nor Disagree
- ◯ Somewhat Agree
- ◯ Agree
- ◯ Strongly Agree

19) I believe autonomous vehicles are designed to operate honestly and truthfully toward users

- ◯ Strongly Disagree
- ◯ Disagree
- ◯ Somewhat Disagree
- ◯ Neither Agree nor Disagree
- ◯ Somewhat Agree
- ◯ Agree
- ◯ Strongly Agree

20) Even when I'm unsure about the autonomous vehicles' decisions, I have faith it is making the right choices

- ◯ Strongly Disagree
- ◯ Disagree
- ◯ Somewhat Disagree
- ◯ Neither Agree nor Disagree
- ◯ Somewhat Agree
- ◯ Agree
- ◯ Strongly Agree
