# Supplementary material for: Remotely seeing is believing: how trust in cyber-physical systems evolves through virtual observation
Source: R Soc Open Sci. 2026 Oct 28;13(10):260220. doi: 10.1098/rsos.260220 (PMC13714369; doi:10.1098/rsos.260220)
Supplement: Supplementary file 1 [file rsos.260220.zip › rsos.260220/rsos260220_si_003.pdf]

Demographics

Please fill out the information below.

1) What is your full name?

____

2) What is your personal email?

____

3) What is your age?

____

4) What is your gender?

- ◯ Male
- ◯ Female
- ◯ Non-binary
- ◯ Prefer not to answer

5) What is your ethnicity?

- ◯ White
- ◯ Black or African American
- ◯ Hispanic or Latino/Latina
- ◯ Asian or Pacific Islander
- ◯ Native American or Alaskan Native
- ◯ Middle Eastern or North African
- ◯ Multiracial or Biracial
- ◯ I do not wish to answer

6) What is your current academic level?

- ◯ Freshman (1st year undergraduate)
- ◯ Sophomore (2nd year undergraduate)
- ◯ Junior (3rd year undergraduate)
- ◯ Senior (4th year undergraduate)
- ◯ Master's student
- ◯ Doctoral student
- ◯ Not a student

7) What are your major(s) and minor(s)? If not applicable, type "n/a"

____

8) What university do you attend? If not applicable, type "n/a"

____

In this survey, Cyber-Physical Systems (CPS) are engineered systems that combine physical (hardware) and computational (software or cyber) components to monitor and control real-world processes. Examples include smart home systems, autonomous vehicles, industrial robots, automated safety systems, etc.

9) Generally, I trust Cyber-Physical Systems

- ◯ Strongly Disagree
- ◯ Disagree
- ◯ Neutral
- ◯ Agree
- ◯ Strongly Agree

10) Cyber-Physical Systems help me solve many problems

- ◯ Strongly Disagree
- ◯ Disagree
- ◯ Neutral
- ◯ Agree
- ◯ Strongly Agree

11) I think it's a good idea to rely on Cyber-Physical Systems for help

- ◯ Strongly Disagree
- ◯ Disagree
- ◯ Neutral
- ◯ Agree
- ◯ Strongly Agree

12) I don't trust the information I get from Cyber-Physical Systems

- ◯ Strongly Disagree
- ◯ Disagree
- ◯ Neutral
- ◯ Agree
- ◯ Strongly Agree

13) Cyber-Physical Systems are reliable.

- ◯ Strongly Disagree
- ◯ Disagree
- ◯ Neutral
- ◯ Agree
- ◯ Strongly Agree

14) I rely on Cyber-Physical Systems

- ◯ Strongly Disagree
- ◯ Disagree
- ◯ Neutral
- ◯ Agree
- ◯ Strongly Agree

15) By checking this box, I certify that I am at least 18 years old and that I give my consent freely to participate in this study.

- ☐ I consent
