# Supplementary material for: Remotely seeing is believing: how trust in cyber-physical systems evolves through virtual observation
Source: R Soc Open Sci. 2026 Oct 28;13(10):260220. doi: 10.1098/rsos.260220 (PMC13714369; doi:10.1098/rsos.260220)
Supplement: Supplementary file 1 [file rsos.260220.zip › rsos.260220/rsos260220_si_004.pdf]

RESEARCH INFORMATION SHEET

Purpose of the Study:
The purpose of this study is to understand how users' perspectives and perceptions of certain technologies may be influenced by how those technologies are portrayed in the media.

Session Overview:

- Before each session, you will receive an email containing instructions and materials required for that specific session.
- During each session, you will watch curated video clips (e.g., of self-driving cars, robotic assembly lines, and power plants) and interact with the application by answering brief questions and pressing buttons in real time.
- There are no right or wrong answers - we are only interested in your impressions and reactions.
- Each session will last approximately 45 minutes in total.
- You can find the general procedure for each session below. In this study, Cyber-Physical Systems (CPS) are engineered systems that combine physical (hardware) and computational (software or cyber) components to monitor and control real-world processes. Examples include smart home systems, autonomous vehicles, industrial robots, automated safety systems, etc. You do not need any prior expertise in CPS to participate.
- Additionally, please review the following icons and their meanings before each session.

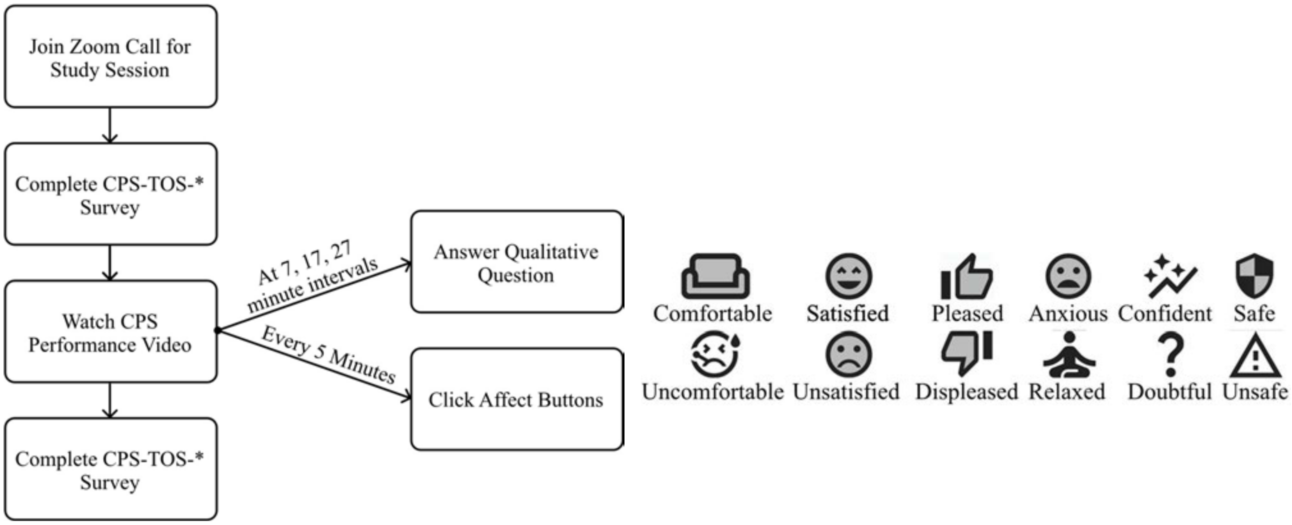

Next Steps:

- You will receive an email containing your session dates and a Zoom link for the first session, along with instructions prior to joining the call.

General Information:

- You will receive a $30 Amazon gift card upon completing your second session, provided you are actively engaged in both sessions. Participation is voluntary, and you may withdraw from the study at any time without penalty; however, full engagement in both sessions is required to receive compensation.
- If you have any questions or concerns about the study, you may contact [redacted] or [redacted].
- We will take all necessary steps to protect your privacy throughout the study. Your personal information will be kept confidential and will not be shared outside the research team unless required by law.
- Any de-identified data will not be used for future research.
- If you have questions about your rights as a research participant, please contact the [redacted]
- Your signature is not required. Your participation in this research is implied if you proceed with the research study.
