# Supplementary material for: Remotely seeing is believing: how trust in cyber-physical systems evolves through virtual observation
Source: R Soc Open Sci. 2026 Oct 28;13(10):260220. doi: 10.1098/rsos.260220 (PMC13714369; doi:10.1098/rsos.260220)
Supplement: Supplementary file 1 [file rsos.260220.zip › rsos.260220/rsos260220_si_005.pdf]

**Introduction**
Hello, and thank you for joining us today. We will begin with the study now.

- At any point during this study, if you encounter issues, please type them into the Zoom chat box.

**CPS-Survey:**
Please take 5 minutes now to complete the CPS-TOS-* survey sent to Zoom chat [insert survey link]
Text "done" in Zoom chat when you have submitted this form.

[Call out when 2 minutes remain; if all participants have finished, begin early]

The 5 minutes are up. If you have not completed the survey, please do so promptly [provide 1-2 minutes longer to complete].

**Setup:**

- I will now share my screen to help guide you through the steps for this study.
- Navigate to the [redacted] web application [paste link in Zoom]. This window should be full-screen.
- Enter the email address you received your Zoom link from. This should be the same email you used to sign up for the study.
- Please click on the session that appears. It should say "Collaborative x".
- You should now see:
    - The starting video
    - The affective response buttons
    - An empty chat window
- If you see a sign-up page instead of this screen, you may have mistyped your email or entered the wrong one. Please return to [redacted] and re-enter your email.
- I will now wait 30 seconds for everyone to get caught up.
- Move your Zoom window so that you can clearly see all of the web application content.
- Click on the sound button once. It should display an "x" to indicate the sound will work for the video.
- I will now stop screen sharing.

**Session Structure:**

- You will complete the session together with the people in this Zoom call. You will be able to see each other's affective responses and qualitative answers in the chat window. No further interaction is required. You may scroll up on the chat if you would like to review your fellow participants' responses.

- Please focus on the video. You may scroll through the button meanings if you need a reminder from the Information Sheet. At certain timestamps, prompts will appear on your screen. I will provide guidance when these prompts show up.

- I will now begin the session. Your video should have started playing automatically. You should see a prompt on the website chat reminding you not to click buttons or type in chat unless instructed. If you experience any issues, please let me know by sending a message in the Zoom chat.

**Post-Session Instructions**

- Please take 5 minutes to complete the CPS-TOS-* survey sent to Zoom chat [insert survey link]. After completing the survey, you are free to leave the Zoom call.

**INSTRUCTIONS**

- Start reading the script at xx:05
- Around 15-20 seconds before a prompt shows up, say:
    - 5-minute mark: In about 15 seconds, a prompt will ask you to click buttons that reflect how you feel about the system's performance and operation. Please click any emotions you have experienced while watching up to this point. The video will pause for 30 seconds and resume playing, during which the buttons will become disabled.

    - 7-minute mark: In about 15 seconds, a prompt will appear asking you to type your answer to a question in the chat. The video will pause for 40 seconds and resume playing. If the video continues before you have finished your response, please take your time to complete it.

    - 10-minute mark: In about 15 seconds, a prompt will ask you to click buttons that reflect how you feel about the system's performance and operation. Please click any emotions you have experienced while watching up to this point. The video will pause for 30 seconds and resume playing, during which the buttons will become disabled.

    - 15-minute mark: In about 15 seconds, a prompt will ask you to click buttons that reflect how you feel about the system's performance and operation. Please click any emotions you have experienced while watching up to this point. The video will pause for 30 seconds and resume playing, during which the buttons will become disabled.

    - 17-minute mark: In about 15 seconds, a prompt will appear asking you to type your answer to a question in the chat. The video will pause for 40 seconds and resume playing. If the video continues before you have finished your response, please take your time to complete it.

    - 20-minute mark: In about 15 seconds, a prompt will ask you to click buttons that reflect how you feel about the system's performance and operation. Please click any emotions you have experienced while watching up to this point. The video will pause for 30 seconds and resume playing, during which the buttons will become disabled.

    - 25-minute mark: In about 15 seconds, a prompt will ask you to click buttons that reflect how you feel about the system's performance and operation. Please click any emotions you have experienced while watching up to this point. The video will pause for 30 seconds and resume playing, during which the buttons will become disabled.
        - There is approximately 5 minutes left in the video, in which you will see two more prompts.

    - 27-minute mark: In about 15 seconds, a prompt will appear asking you to type your answer to a question in the chat. The video will pause for 40 seconds and resume playing. If the video continues before you have finished your response, please take your time to complete it.

    - 30-minute mark: In about 15 seconds, a prompt will ask you to click buttons that reflect how you feel about the system's performance and operation. Please click any emotions you have experienced while watching up to this point. The video will pause for 30 seconds and resume playing, during which the buttons will become disabled.
        - Stop the video **after** they have finished clicking buttons. Ask them to return to the Zoom call to complete the post-survey.
